# Sex differences in the neural mechanisms mediating addiction: a new synthesis and hypothesis

**DOI:** 10.1186/2042-6410-3-14

**Published:** 2012-06-07

**Authors:** Jill B Becker, Adam N Perry, Christel Westenbroek

**Affiliations:** 1 Molecular and Behavioral Neuroscience Institute, University of Michigan, Ann Arbor, MI 48109, USA; 2 Department of Psychology, University of Michigan, Ann Arbor, MI 48109, USA; 3 Neuroscience Program, University of Michigan, Ann Arbor, MI 48109, USA; 4 Department of Psychiatry, University of Michigan, Ann Arbor, MI 48109, USA

**Keywords:** Addiction, Dopamine, Acetylcholine, Norepinephrine, Dynorphin, Cocaine, Heroin

## Abstract

In this review we propose that there are sex differences in how men and women enter onto the path that can lead to addiction. Males are more likely than females to engage in risky behaviors that include experimenting with drugs of abuse, and in susceptible individuals, they are drawn into the spiral that can eventually lead to addiction. Women and girls are more likely to begin taking drugs as self-medication to reduce stress or alleviate depression. For this reason women enter into the downward spiral further along the path to addiction, and so transition to addiction more rapidly. We propose that this sex difference is due, at least in part, to sex differences in the organization of the neural systems responsible for motivation and addiction. Additionally, we suggest that sex differences in these systems and their functioning are accentuated with addiction. In the current review we discuss historical, cultural, social and biological bases for sex differences in addiction with an emphasis on sex differences in the neurotransmitter systems that are implicated.

## Introduction

**Figure 1:**
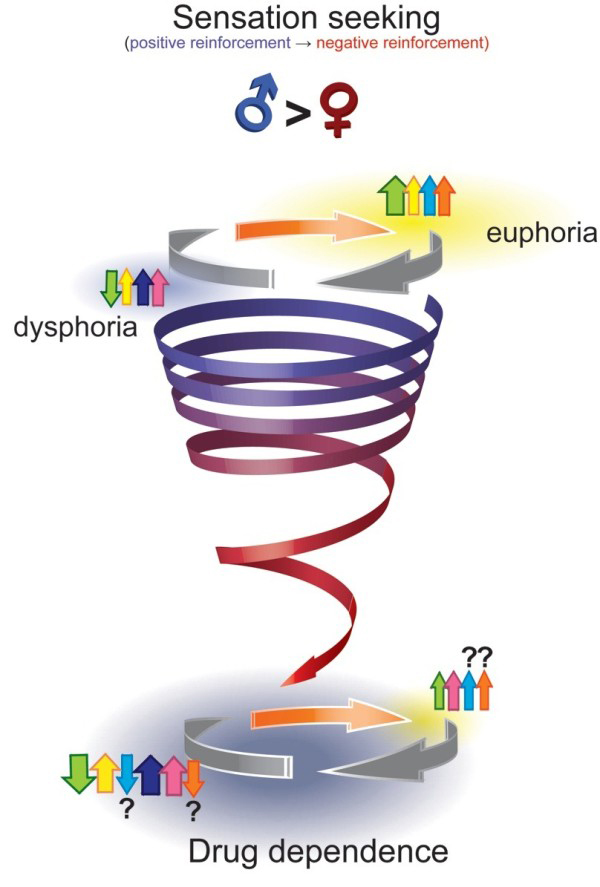
**The downward spiral from sensation-seeking into addiction.** The spiral depicts individuals initiating drug use (large orange arrow) primarily due to positive reinforcement (i.e., seeking the hedonic effects of drugs, such as euphoria, increased energy and alertness, or “the thrill,” which are indicated by yellow shading), which are attributed to acute increases in dopamine (DA, green arrow), norepinephrine (NE, yellow arrow), endogenous opioids (ENK/END/EM, light blue arrow), and acute increases in acetylcholine (ACh, orange arrow). A post-intoxication “crash” follows these acute positive effects due to an “over-correction” by compensatory mechanisms leading to a transient dysphoria (blue-grey shading), which is largely attributed to reduced DA function, ongoing NE activity, and increased dynorphin (DYN, dark blue/purple arrow) and corticotropin releasing factor (CRF, pink arrow) signaling. Neurochemical function and affective state eventually normalize during drug-free periods (white shading between grey arrows). Following repeated use, drug-induced adaptations can also result in the development of psychopathologies and physical symptoms that further reinforce drug use out of negative reinforcement (as depicted by the transition in the spiral from blue to red). A larger proportion of men compared to women may initiate drug use for their positive effects. However, sex differences in the highlighted neurochemical systems may also lead to different trajectories from sensation-seeking toward dependence in men and women. (The magnitude of neurochemical responses is indicated by the relative sizes of the arrows, refer to text for details on sex differences). Modified from Koob and Moal [1].

The path from initial drug use to addiction is often described as a downward spiral [1]. The euphoria of first use deteriorates with habituation, to be replaced with heightened incentive salience associated with the drug and the cues that predict the drug as well as dysphoria in the absence of the drug. This is followed by addiction, compulsive craving for the drug, and exacerbation of dysphoria with drug withdrawal. While this narrative may capture the changing relationship between users and their drugs over time, it fails to recognize the diverse reasons contributing to initiation of drug use, which may ultimately influence how quickly an individual develops addiction. For many, illicit drugs are initially taken for their positive reinforcing effects, such as feelings of euphoria, energy, focus or sexual enhancement (Figure 1). For many other individuals, illicit drug use is initiated primarily to self-medicate another condition (i.e. negative reinforcement), such as depression, anxiety, chronic pain or post-traumatic stress disorder (PTSD), just to name a few. Thus, in these latter individuals, drug use provides temporary relief and functions as a maladaptive coping strategy to deal with the alterations in reward-related processes and affective state that characterize each psychopathology.

While the basic neural systems involved in positive and negative reinforcement, are similar in males and females, sex differences are present in how these neural systems are organized, activated and connected with the rest of the brain, and these are postulated to underlie sex differences in the path to addiction. Additionally, sex differences in these systems and their functioning are accentuated with drug use and the progression towards addiction. There is an extensive body of literature concerning the neural systems contributing to the development of addiction. In general, the monoamine systems (e.g., dopamine (DA) and norepinephrine (NE)), neuropeptides (e.g., corticotropin-releasing factor (CRF) and the endogenous opioid peptides) and others (e.g., acetylcholine (ACh)) have been shown to participate in either the rewarding effects of abused drugs or their negative effects observed during withdrawal. With the exception of the DA system [2-4], little attention has been paid to sex differences in these other systems and how they might differentially contribute to the risk of addiction in males and females.

It is the thesis of this review that sex differences exist along every aspect of the spiral pathway towards addiction. In addition, we propose the existence of a second “steeper” spiral, for which initiation of drug taking occurs to alleviate self-perceived symptoms of (stress-related) psychiatric disorders. There are sex differences in why men and women enter onto the path that can lead to addiction. Men and boys are more likely to engage in risky behaviors that include experimenting with drugs of abuse primarily for their positive reinforcing effects. In susceptible males, they are drawn into the spiral that can eventually lead to addiction. Women and girls are more likely to take drugs to reduce stress or alleviate psychological distress (e.g., depression and PTSD), thus they enter into the downward spiral already burdened with neurological changes that may promote their transition to addiction more rapidly. We propose that neither mode of entry into the spiral is exclusive for men or women, but rather, different proportions of men and women enter through the two spirals. Our thesis is that in the presence of stress-related psychopathologies, or border-line disorders, the transition from drug use to dependence will be faster. In addition, sex differences in the underlying neurobiological mechanisms of these disorders interact with the effect of drugs of abuse to result in sex differences in the consequences of drug use and abuse in a more vulnerable population.

We begin with an historical overview of evidence for sex differences in addiction and drug abuse in humans. This is followed by descriptions of the effects of drugs of abuse with initial drug use, consequences of chronic drug use and several of the brain systems involved. Sex differences will be illustrated throughout each section. We will focus mostly on the psychostimulants and opiates.

We hope this review will accomplish at least two objectives: 1) highlight the seemingly ubiquitous presence of basal sex differences in nearly every system implicated in addiction, and 2) reveal the shocking lack of knowledge of how these differences contribute to divergent (or similar) responses to drugs of abuse and the development of addiction in males and females.

### Historical background

The history of use of psychoactive drugs is found throughout our recorded history. One of the first recorded uses is in the Odyssey by Homer from the 9^th^ century B.C. where Helen, daughter of Zeus, gave a drug that is thought to be opium to Thelamachus and his men so they could forget their grief at Odysseus’ absence, "it entered into Helen’s mind to drop into the wine that they were drinking an anodyne, mild magic of forgetfulness. Whoever drank from this mixture in the wine bowl would be incapable of tears that day… The opiate of Zeus’ daughter bore this canny power. It had been supplied her by Polydamna, mistress of Lord Thon in Egypt…" p. 59 [5]. Of course this passage records the use of both wine (alcohol) and opium, pointing out that men and women have also been using and abusing alcohol throughout our history, as well as that opium was traded even during the time of Odysseus.

It is difficult to determine the extent that women were using or becoming addicted to drugs throughout the centuries, since most of recorded history focuses on men. We know that men used drugs and became addicted, but to what extent and under what conditions have women suffered from addiction in the past? Quantitative data on the number of people (men or women) addicted to any drug do not exist before late in the twentieth century. Qualitative data about the causes of drug use and addiction are colored by perceptions of the roles of men and woman in society at the time. We relate the historical evidence for drug use by women, to contextualize the differences in the path to addiction for women vs. men.

What we know of historical patterns of drug use comes mostly from the United States where attempts to document use in both men and women began in the 1800 s, although there are anecdotal reports as well as characters in literature that contextualize drug abuse in other cultures throughout the years, as illustrated by the quote from The Odyssey above. More recently, the United Nations Office on Drugs and Crime has published annual reports on drug production and use throughout the world, but these reports are not stratified by sex/gender, and there are large populations for which data are missing. For example, in China and Africa there are no data on drug use among school age individuals [6].

#### Prevalence of drug use in women

##### Ethanol

The use of alcohol is recorded throughout history as an anesthetic, antiseptic and medication, as well as for its use in beverages. In plays of the ancient Greeks, women were frequently depicted as intoxicated, and drinking wine was linked to sexual promiscuity in women [7]. Throughout the ages ethanol was used by both men and women, but brewing was considered a female domestic trade in Germany, until the late 1600 s when the trade became taxed and then men took over the practice [7]. In England in the 1700 s the overuse of distilled alcohol became a “woman’s problem”. Prior to the 1700 s most alcohol consumed in England was ale, beer or wine, and these tended to be consumed more by men, perhaps because of the establishments in which spirits were consumed. With the arrival of distilled liquors in the 1700 s, gin became known as the woman’s drink, which fueled the ‘gin craze’ in England. Women were selling gin and drinking gin, gin was sold in places where women congregated and here women were selling gin to other women. While it is difficult to get quantitative data from the eighteenth century, there was a clamp-down on gin sales in 1738 and 75% of the gin-sellers appearing before a magistrate were women [8]. Female use of gin was made a social problem with a number of highly publicized cases that lead to tighter restrictions and higher taxes on gin, which further reduced use for both men and women, but women in particular.

##### Opiates

As discussed in Brownstein (1993), the cultivation of poppies to obtain opium began with the Sumerians living in what is now Iraq during the third century B.C., although opium was being obtained from poppies before that time. From the literature one can trace to the 13^th^ centuries the trading of opium in Europe and Asia minor, and reports of addiction can be found in manuscripts from the 16^th^ and 17^th^ centuries [9]. The British went to war with China in 1839 and again in 1856 for the right to continue to sell their opium produced in India to China, these became known as the First and Second Opium Wars. China lost both wars and was forced to open its borders to unrestricted trade and to permit non-Chinese individuals into mainland China [10]. The active ingredient in opium was isolated in 1806 and was called morphine after the god of dreams Morpheus [9]. With the isolation of the active compound, more uses for the drug were introduced, and both doctors and pharmacists dispensed medicines that contained opiates for a multitude of ailments resulting in addiction for men and women [11].

The best quantitative data of a historical nature on women and drug abuse are on the opiates and solutions containing opiates. In the 1800 s it was widely acknowledged that in the USA more women than men were addicted to opiates (opium, morphine, laudanum or heroin). This sex difference in opiate use was quite dramatic with estimates ranging from 66% to 80% of the opium users being women during the late 1800 s [12]. As discussed by Kandall (1999) it was hard to get an accurate estimate of the number of women who were addicts in the 1800 s, because women tended to use opiates clandestinely, self-medicating with doses that allowed them to continue to function. In fact women frequently used opium for years without the knowledge of their husband, friends or family. Many physicians considered opium addiction among women to be an upper class affliction, but in fact, when doctors were surveyed in a more systematic manner there was no distinction by class, occupation or regional location. Housewives, prostitutes, women in rural farming communities, women living in Massachusetts or Alabama, all were more susceptible to opiate addiction than were their male counterparts. This was in large part because physicians and pharmacists freely over prescribed and dispensed legal opiates [12]. Furthermore, the readily available patent medicines contained large amounts of opiates and alcohol, and this contributed to their widespread use throughout the country. Doctors at the time concluded that, “women were more prone to opium addiction because of their ‘more nervous organization and tendency to hysterical and chronic diseases’” (Hamlin, 1882, as cited in Kandall 1999, p.29).

By the early 1900s there were approximately 50,000 opiate-containing patent medicines available in the USA [13]. Then, in 1906 the Pure Food and Drug Act was passed, and this law is credited with resulting in a dramatic decrease in drug addiction throughout the country. This is the law that created the Food and Drug Administration (FDA) which required that the FDA approve drugs intended for human consumption, that certain drugs be available only by prescription, and that drugs that were habit forming needed to be labeled as such (Pure Food and Drug Act of 1906, United States Statutes at Large (59th Cong., Sess. I, Chp. 3915, p. 768–772)). This law effectively put the patent medicine industry out of business, since when these medicines were tested they were not approved for sale by the FDA. Then, in 1914 the Harrison Tax Act was passed, and this law effectively limited the non-medical use of narcotics (opium, morphine and its various derivatives, and the derivatives of the coca leaf including cocaine) by imposing a prohibitive tax on all non-medical sales of these drugs^1^. All over the country, the ready access to addictive patent medicines was eliminated and drug addiction in both men and women declined. For women, the result of the Harrison Tax Act was that the proportion of women narcotic addicts declined to 50% of the population by 1918, and the proportion continued to decline until women were approximately 25-30% of addicts in the USA by the beginning of World War II [13].

This example, considered with the use of ethanol in the 1700 s in England, illustrates that when social conditions allow for easy access to a drug of abuse women can be more likely to escalate use to addiction than are men, perhaps due to the tendency to self-medicate. When restrictions are tightened, use by women falls off. On the other hand, one must also take into consideration the position of women in society during these times. Women were without a profession, frequently left alone, men of the times tended to value a frail and retiring personality in their women, so self-medication to alleviate physical and psychological conditions was tolerated and to some extent encouraged [12].

##### Other drugs

After World War II women who had been busy and employed during the war returned to the role of homemaker, and physicians began to prescribe the use of tranquilizers and sedatives to alleviate the stress and tension of the discontented housewife [12,13]. By the end of the 1960s two-thirds of the prescriptions for the tranquilizers Valium and Librium were to women. The drug industry at the time was also promoting the use of amphetamines as appetite suppressants, and women were consuming 80% of the prescription amphetamines in the USA. Since the 1960s the use of all addictive drugs by women has been increasing. It is still the case, that for individuals 18 or older, there are more men than women who are drug addicts. In the 2008 SAMSHA report, among youths aged 12 to 17, however, the rate of substance dependence or abuse among males was similar to the rate among females (8.0 vs. 8.1%) [14].

In the college age population surveyed in the 2008 SAMSHA report, containing both individuals attending college and an age-matched cohort, use of amphetamine was a little higher among college males (7.2%) than college females (4.8%), but somewhat lower among males in the non-college segment (6.3%) than among non-college females (7.7%). Cocaine showed a similar profile with college females (3.0%) reporting less annual cocaine use than college males (6.4%), and non-college males and females both reporting higher rates of cocaine use (9.3% and 8.7% respectively). For Vicodin (an opiate-based prescription medication) in 2008 males and females showed the same usage rate: 6.7% vs. 6.6%. Non-college females reported the highest rates of use for both sedatives (barbiturates) and tranquilizers, relative to all other groups. Alcohol use was approximately the same in males and females, with males having higher prevalence of binge drinking than females. The annual prevalence of marijuana use did not differ significantly between males and females among college students, nor among the non-college respondents [14].

The historic prevalence and pattern of drug abuse among males and females highlights a couple of points. First, availability of opiate drugs in the 1800 s and sedatives or tranquilizers in more recent times has lead to greater abuse of these drugs by women than men due to a combination of factors including physician’s recommendations, self-medication, and other social factors such as lack of education and job status (unemployed, homemaker, etc.). Since the 1960s the psychomotor stimulants (amphetamine, methamphetamine, and cocaine) have been used by women for appetite suppression and as a ‘pick-me up’. During the 1980s and 1990s the illicit use of these drugs among individuals under the age of 25 was predominantly by males. Since 2004, however, the sex difference in use of these drugs has declined and there has been no difference between males and females in stimulant use among this age group in recent surveys [14]. Overall, availability of drugs coupled with dissatisfying social conditions, stress, anxiety, and depression tends to exacerbate drug abuse and addiction in women. While such conditions can also increase drug use in men, it is our hypothesis that on the average this happens more often in women.

Finally, comorbidity of psychiatric disorders and substance abuse is substantial, 30-41% of subjects with a lifetime drug use problem suffer from at least one mood or anxiety disorder, and these associations are stronger in women than in men [15-17], supporting the idea that self-medication for mood disorders is a major path to addiction in women. While there are clearly cultural and social factors that impact whether a woman vs. a man will take an illicit drug and then continue to take the drug to the point of compulsive use and/or addiction, there are also biological sex differences that contribute. Furthermore, the same patterns of more rapid acquisition and escalation of drug use are seen in female rodents compared with males. We make the case that this is due to sex differences in the neurobiology of the system in this review.

### Patterns of drug taking behavior in women and men

Only a small percentage (16-17%) of people who use drugs will progress to a state of dependence [18,19]. Sex differences have been reported in the risk of progression to dependence for several types of drugs. For example, males have a higher risk for cannabis and alcohol dependence, whereas for cocaine the risk is equal for men and women [19].

Substance abuse and dependence are both characterized by maladaptive patterns of substance use that lead to clinically significant impairment or distress (DSM-IV, [20]). The criteria that must be met for ***abuse*** include substance use that leads to problems at work, physically hazardous situations, and legal problems and/or interpersonal or social problems. The specific criteria for ***dependence*** include tolerance, symptoms of withdrawal, escalation of intake, persistent unsuccessful desire to control substance use, considerable time spent in activities to obtain or use the substance, other previously valued activities are reduced because of substance use, and substance use continues in the presence of adverse consequences [21]. When a user transitions from recreational to compulsive drug use, there is an increase in the amount of drug used daily, primarily from an increase in frequency of use rather than increases in the dose (for review [22]).

More men than women meet criteria for drug abuse and dependence, and men show a higher prevalence for dependence on alcohol and marijuana. On the other hand, even though more men than women use cocaine and psychotherapeutics, more women show dependence for these substances [23,24].

Prevalence of drug abuse and addiction is only one index of how males and females differ in their responses to drugs of abuse. Other characteristics of drug abuse are also sexually dimorphic, including age of drug use initiation, rate of escalation of drug use, and quantity of drug consumed. This is particularly true for the psychomotor stimulants [2,25], but is also true for other drugs of abuse [4]. For example, women start using cocaine or amphetamine at an earlier age than do men, the rate of drug use escalation is greater for women than for men, and when women seek treatment they are consuming greater quantities than are men [2,25]. In addition, women report higher craving then men, and exhibit more medical problems [16]. Although no sex differences in these aspects of drug addiction have been reported as well [19,26].

Why men and women or boys and girls begin using drugs is also different. The best data are for consumption of alcohol, but the same pattern of results is found for other drugs of abuse. In general, males report starting drug use for the thrill or to enhance their behavior in social situations, while girls report drug use to enhance their ability to cope with bad feelings, reduce stress, and decrease feelings of social isolation [27,28]. This sex difference in drug use initiation is also seen in the drugs that are initially used. For opiate addiction, males will tend to use heroin or other street opiates, while females tend to use prescription opiates first and then progress to the use of narcotics obtained without a prescription [29]. Perhaps as a consequence, heroin use is more common in men, whereas women are more prone to use other opiates, barbiturates, sedatives, and amphetamines.

Importantly, childhood abuse and neglect predicts subsequent illicit drug use in adulthood in women, but not men [30]. In women, drug use following early abuse appears to manifest as part of a generalized problem behavior syndrome that includes prostitution, homelessness, delinquency, criminal behavior and problems at school [30]. This relationship between early abuse and subsequent drug use may reflect the sex difference in the reason for drug use initiation, since women tend to be self-medicating for feeling of social isolation and stress reduction.

If one considers individuals who are incarcerated, the prevalence of substance abuse and dependence among incarcerated women is higher than for their male counterparts [31,32]. This may reflect the fact that the overall number of women in prison is much lower than men, and their convictions are often due to drug-related crimes. When examining participants with drug-related offenses that were placed in drug court programs, it is clear that women began using alcohol and marijuana at later ages, but tended to initiate cocaine use earlier than men. Additionally, while the time between initiation and the development of problematic use was similar for men and women for alcohol and marijuana, problematic cocaine use occurred much earlier in women [33].

### Sex differences in the acute/subjective effects

#### Humans

With the exception of the stimulants (e.g., cocaine and amphetamine), the subjective effects of most drugs do not differ between men and women (reviewed in [34]). In the case of cocaine and amphetamine (AMPH), men often (but not always) report greater subjective effects than women [35]. This difference is most likely due to the fact that the subjective effects of stimulants vary over the course of the menstrual cycle, with men and women in the follicular phase (when E2 levels are low at first and rise slowly; P levels are low) being more similar to one another than either is to women during the luteal phase (when E2 levels are moderate and P levels are high).

There is substantial evidence that sex differences in the response to stimulants are due in large part to the fluctuations in estrogen (E2) and progesterone (P) that occur over the female reproductive cycle. For example, several of the positive subjective effects of AMPH, such as euphoria, desire, increased energy and intellectual efficiency, are potentiated during the follicular phase relative to the luteal phase [36]. Additionally, administration of exogenous E2 during the follicular phase further increases the subjective effects of AMPH [37].

In contrast to E2, the subjective effects of psychostimulants are negatively correlated with salivary P levels in women [38]. Additionally, exogenous P attenuates many of the positive subjective effects of cocaine when administered to women during the follicular phase, but has negligible effects in men ([39], but see [40,41]). Conversely, exogenous P also has been shown to increase the positive subjective effects of AMPH in women [42].

The role of androgens (e.g., testosterone, T) in the pattern of drug use in men and women has received far less attention than E2 and P. Similar to E2, T concentrations vary over the menstrual cycle [43]. Additionally, there are circadian and seasonal rhythms to T concentrations [44-47]} and several types of social experiences can modulate plasma T profiles in both sexes, including sexual arousal, winning/losing sports competitions, exposure to an infant’s cries and providing nurturing care [48-57]. T concentrations can be modulated by social experiences as well as by exogenous drugs in both males [58,59] and females [60]. Thus, the relationship between the response to drugs of abuse and circulating T is dynamic and bi-directional.

Taken together, these results suggest that if women start taking drugs such as cocaine or amphetamine to self-medicate for depression or anxiety, the stage of menstrual cycle may impact both their subjective to mood to begin with, as well as the efficacy of the drug to overcome the endogenous state. This could affect the risk for transitioning from use to dependence. It is beyond the scope of this review to cover all of psychoactive drug-induced changes in endocrine responses. We will focus on what is known about sex differences in the effects of drugs on E2, P, T and corticosterone/cortisol (CORT) in the following sections.

#### Animal models

In preclinical models, the subjective effects of drugs are often examined in the conditioned place preference (CPP) paradigm [61]. Female rats develop CPP to lower doses of cocaine than do males [62,63]. Yet both sexes show equivalent CPP at higher doses of cocaine. Reinstatement of CPP is also more pronounced in females at higher cocaine doses [64]. The strength of CPP in females is highly dependent upon ovarian hormones. Cocaine CPP is attenuated in ovariectomized (OVX) females, whereas treatment with both E2 and P (but not E2 alone) potentiates CPP [65]. No sex differences in CPP to (meth)AMPH have been reported in studies using intact males and females [66,67]. AMPH does not induce CPP in OVX females unless they are treated with E2 or E2 and P, an effect apparently mediated by estrogen receptor β (ERß) [68].

Sex differences in the rewarding properties of morphine have been reported, however, there are differences among various rat strains. In Wistar rats, females find lower doses of morphine more rewarding than males do [69]. In Sprague–Dawley rats, however, there is no difference in morphine CPP between males and females at lower doses, but females continue to display CPP at high doses of morphine that males do not prefer [70]. Thus, sex differences in the rewarding value of drugs of abuse (as indicated by CPP) vary with the drug, the dose and the hormone condition of the female rodent.

### Drug taking behavior- effects of gonadal hormones

The amount taken and frequency of drug use are often different in men and women. In a survey of heroin addicts, men and women reported a similar frequency of heroin and alcohol use in the past 30 days; however, women reported significantly more days of cocaine use [71]. Similarly, in another survey of men and women in a treatment center, women reported higher frequencies for lifetime and current (i.e., past 12 months) use of crack/cocaine than men, whereas there were no differences in the frequencies of alcohol, marijuana or heroin use [72]. When examining sex differences in stimulant users that primarily abuse crack or powder cocaine, women reported greater frequencies of crack use than men, whereas patterns of powdered cocaine use were similar between the sexes [73]. Women also appear to be more vulnerable to escalation of drug use and show a faster transition from initial use to dependence [74,75].

#### Ovarian hormones

Evidence from studies in both humans and animals indicate that ovarian hormones modulate self-administration of stimulants and thus may influence sex differences during different phases of cocaine addiction. E2 administration to ovariectomized females affects many psychostimulant drug-induced behaviors, including self-administration [76-84]. For example, Hu et al. (2004) found that in ovariectomized female rats, exogenous E2 treatment alone was sufficient to facilitate acquisition of cocaine self-administration. E2-facilitated cocaine self-administration has also been found in other studies [84,85]. Finally, acquisition of cocaine self-administration is markedly reduced by ovariectomy, and restored by E2 replacement [86]. Sex differences and the effects of E2 are not limited to psychostimulants, and E2 has been found to also facilitate acquisition of self-administration of opioids in ovariectomized rats; females acquire faster and show a higher motivation to self-administer morphine and heroin than males [87-89]. Furthermore, there is no effect of castration of male rats on acquisition of cocaine self-administration behavior and a dose of E2 that enhances self-administration in female rats has no effect on cocaine self- administration behavior in males [90]. Thus, the effects of E2 on the acquisition of cocaine self-administration are sexually dimorphic.

Female rats will work harder for cocaine during the estrous phase of the cycle than during other phases of the cycle, and females work harder than male rats [85]. The finding that the motivation to self-administer cocaine is greater during the estrous phase of the cycle may be related to the finding that stimulant-induced DA release is enhanced during estrus relative to diestrus [91,92]. In contrast, sucrose self-administration does not vary across the estrous cycle [93], suggesting that drug taking behavior taps into a slightly different motivation circuit or that drugs of abuse are more effective at activating these neural circuits and so effects of the estrous cycle are observed.

Female rats also ‘binge’ for a longer initial period of time, take more cocaine over a 7-day access period, and show a greater loss of diurnal control over cocaine intake than do males [94]. When the role of E2 in ‘binge’ cocaine intake and subsequent motivational changes is examined, E2 treatment increases the initial binge length and enhances cocaine self-administration [95].

Patterns of hormone secretion are also altered during withdrawal from cocaine. During the first month of cocaine abstinence, cocaine-dependent women show elevated CORT and progesterone concentrations across their cycle compared to healthy controls [96]. In this same study, the authors report a reduction in negative affect at the end of the luteal phase, which may relate to findings in another study that a majority of women entering treatment for cocaine dependence were currently in the early follicular phase and were more likely to be experiencing high levels of anxiety [97]. High levels of anxiety and depression at the start of treatment are also associated with increased cocaine-positive urine tests at intake, which in turn predicts treatment retention [97]. Thus, fluctuations in hormones and mood over the menstrual cycle, which are both influenced by drug use and withdrawal, may impact patterns of drug taking, and the likelihood of entering and successfully completing treatment programs in women.

#### Testosterone

As described previously, androgen secretion in males is often dynamically regulated by social interactions that impact status or reproduction [48,54,98-101]. These fluctuations in androgens over the course of the day may influence the pattern of drug use and potential for relapse in men. The latter may be especially relevant to several drug-seeking triggers and may contribute to the overlap between sexual activity and drugs (especially as seen with the stimulants) [102-105]. Using real-time electronic diaries to track mood, drug craving and use, participants increasingly endorse “was in a good mood” prior to cocaine use, but not craving [106]. This is in contrast to heroin craving, which was more often preceded by negative feelings (e.g., felt “angry,” “worried,” or “sad”), among others [106]. In fact, men report relapse following positive feelings as well as a connection between feeling good (e.g., from winning at sports) and drug craving. Conversely, opiate and cocaine addicts are reported to have reduced T concentrations during withdrawal or while on methadone maintenance [107,108].

### Aspects of addiction-criteria modeled in animals

Escalation of drug intake is one characteristic of addiction. The rate of drug use escalation is greater for women than for men, and when women seek treatment they are consuming greater quantities than are men [2,25]. The escalation of drug intake has been extensively studied in male rats. In these studies, animals that have daily access to drugs for only 1–2 h rapidly stabilize their drug intake, while animals exposed to extended daily access (>6 h/day) show increasing drug-intake over time [109-111]. Other reinforcement schedules can also lead to escalation in drug taking [111-113]. Thus, environmental conditions can influence drug-taking behavior and the preclinical situation can be made to reflect the clinical pattern of drug taking. Most of this research has been has been conducted in male rats, but females are more likely to escalate drug taking and show dysregulated intake after extended daily access [114], an effect that is modulated by gonadal hormones [115].

Persistence of drug taking/seeking in the face of adverse consequences is an aspect of addiction that is not often investigated in animal models. Rats with limited drug experience will stop responding for drugs when the drugs are paired with a stressor like a foot shock. After extensive drug taking exposure some rats will continue to respond for the drug, even when receiving a foot shock, which is thought to be related to compulsive drug taking as observed in human addicts [116,117]. To our knowledge no data are available for females in this regard.

Finally, that fact that only a small percentage (16-17%) of people who have used cocaine or other drugs that are abused develop dependence [18] is largely ignored in preclinical addiction research. During preclinical drug self-administration studies, the drug is easily obtained without much effort or risk on the part of the rat, so all animals learn to self-administer with stable levels of responding. When multiple addiction-like traits (i.e., high motivation to take drugs, persistence of drug seeking when no drugs are available, and resistance to negative consequences) are examined within a population of rats, only a small percentage of animals (<20%) meet all three criteria, and only after long-term chronic self-administration [118,119]. This indicates that chronic drug exposure is necessary to develop addiction-like behavior in rats. It is unknown whether the same proportion of female rats would meet the three addiction-like trait criteria.

### Stress and craving/reinstatement of drug seeking

Sex differences in stress-reactivity are extensively reported, both in the hypothalamic-pituitary adrenal (HPA)-axis response and neurobiological consequences of stress in the brain. As reviewed recently, the relations between drug abuse and sex differences in the stress system are compelling [120]. For example, activation of the HPA-axis occurs with the administration of many different types of drugs of abuse [121-123], with females showing an enhanced stress response to cocaine [124]. Conversely, stress affects several aspects of drug-taking behavior [125-127], and activation of different components of the HPA-axis are essential for acquisition and maintenance of self-administration of cocaine [128], motivation to self-administer cocaine, sensitization to cocaine, and CPP for cocaine but not morphine [129].

Exposure to stressors induces craving in abstinent drug users [127,130-133] and thus stress plays an important role in maintenance and relapse of substance abuse. Beside stressors, exposure to cues associated with drug use also result in craving. Interestingly, these cues activate the HPA-axis and induce anxiety and subjective feeling of stress [133-135], indicating cues act as stressors as well. Sex differences have been found to cue and stress-induced craving. Cues associated with drugs and stress both increase drug craving in men and women; however, women appear to have a greater craving response and appear to be more sensitive to the effects of stress [130,133,134]. Neurobiological differences have also been found, with women addicted to cocaine showing a greater reactivity to cocaine-associated cues than men and a reduction in glucose metabolism in frontal cortical areas, suggesting an impaired cognitive control after exposure to cocaine-cues [136,137]. Interestingly, corticostriatal-limbic hyperactivity was linked to stress-cues in women and drug-cues in men, indicating a differentially activated, but overlapping, circuitry for craving in men and women [137]. The impact of cues and stress on craving appears to depend on the stage of the menstrual cycle, with decreased craving and anxiety being associated with higher P levels during the midluteal phase [138].

Reinstatement of cocaine-seeking in rats is used as a model for cocaine-craving in humans. Estrous females show greater responding on the first day of extinction training when the reward is no longer available and show greater cocaine-induced reinstatement of cocaine-seeking compared to proestrous and diestrous females, which is associated with low levels of P [139]. E2 affects cocaine-induced reinstatement in ovariectomized animals and augments cocaine-seeking. Sex differences in the amount of cocaine-seeking during the first days of extinction training have been reported, with females displaying greater amounts of seeking than males, and there are also effects of the estrous cycle on cue-induced reinstatement [140,141].

In rats (similar to in humans), stress enhances self-administration and reinstatement of drug-seeking for several types of drugs of abuse in both males and females [142]. Sex-differences in the effects of stressors on reinstatement are also seen in rats, with females having greater stress-induced reinstatement of cocaine seeking than males. In addition, the estrous cycle modulates the effects of stress, with proestrous females displaying higher levels of stress-induced reinstatement of cocaine-seeking [140,141].

### Underlying neurobiology

**Figure 2:**
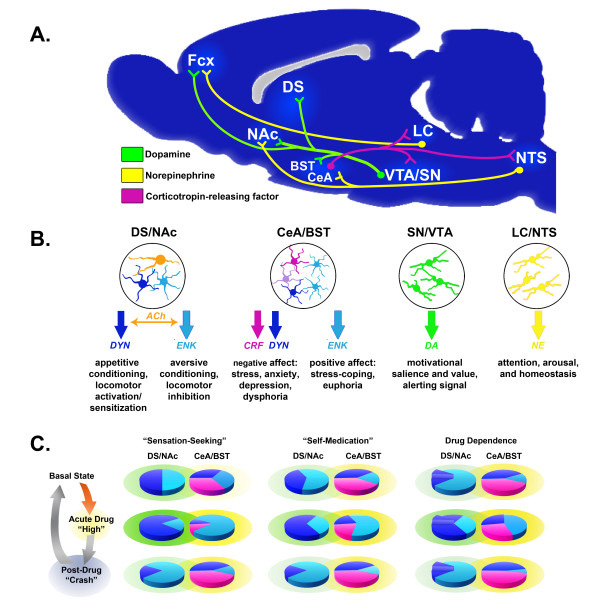
**The central pathways of addiction and their associated neurochemical systems. A.** Sagittal rat brain section depicting the systems involved in reward/aversion and addiction. **B.** Medium spiny neurons (MSN) are the primary sites of synaptic integration in the DS/NAc, which regulate locomotion and reward processes. Striatonigral MSN (DYN), are essential for the reinforcing effects of drugs, whereas striatopallidal MSN (ENK) oppose the actions of striatonigral MSN and promote aversion. Cholinergic interneurons provide ACh in the DS/NAc, which is critical for regulating the balance between striatonigral and striatopallidal MSN (among other functions). The CeA/BST contain several neuron types expressing neuropeptides (and other neurotransmitters). CRF and DYN neurons contribute to negative affect, whereas ENK neurons contribute to positive affect. DA neurons in the SN/VTA send projections throughout the forebrain, which convey motivational salience and value, as well as providing an alerting signal for stimuli with potential significance. NE neurons in the LC/NTS send projections throughout the forebrain, which enhance attention and arousal and modulate systems critical for maintaining homeostasis. **C.** The cycle of drug abuse/withdrawal alters the balance of signaling in the DS/NAc and CeA/BST. Individuals initiating drug use primarily for “sensation-seeking” (refer to Figure 1) or “self-medication” (refer to Figure 5) have different neurochemical profiles in the basal state and during acute intoxication and the post-drug “crash.” The relative size of the pie pieces (e.g., DYN, ENK and CRF) indicates the predominance of each system, whereas the shading density (e.g., DA and NE) indicates the relative extracellular monoamine concentrations. Neurochemical profiles are further altered during dependence, with plasticity mechanisms in the DS/NAc potentiating striatonigral circuits driving compulsive drug-seeking behavior (indicated by the raised pie wedge). The collective neurochemical changes and their associated effects on DS/NAc and CeA/BST neurotransmission contribute to more frequent cycles of abuse and relapse that are the hallmarks of the spiral to addiction (refer to Figures 1 and 5).

The neural circuitry contributing to drug use and the development of addiction has been the subject of several recent reviews [143-146]. Therefore, we will only provide a brief overview to provide context for the sex differences described in the following sections. The vast majority of research regarding the effects of abused drugs and the neural changes underlying dependence has been focused on neurotransmission within and between the frontal cortex (Fcx), nucleus accumbens (NAc), dorsal striatum (DS), central nucleus of the amygdala (CeA) and bed nucleus of the stria terminalis (BST) (Figure 2).

**Figure 3:**
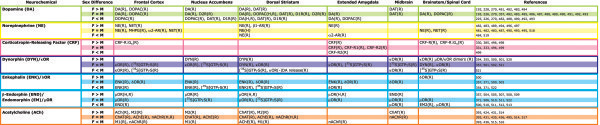
**Sex differences in basal neurochemical systems involved in reward, aversion and addiction.** A summary of the published information about sex differences in the neurochemistry of the reward system appears in this figure [193,225,226,228,269,329,330,332,353,354,356,370,384,392,423,430,434,435,480-521]. Abbreviations are as follows, (R): data collected from rodents; (H) data collected from humans M: male, F: female, for other abbreviations see list.

**Figure 4:**
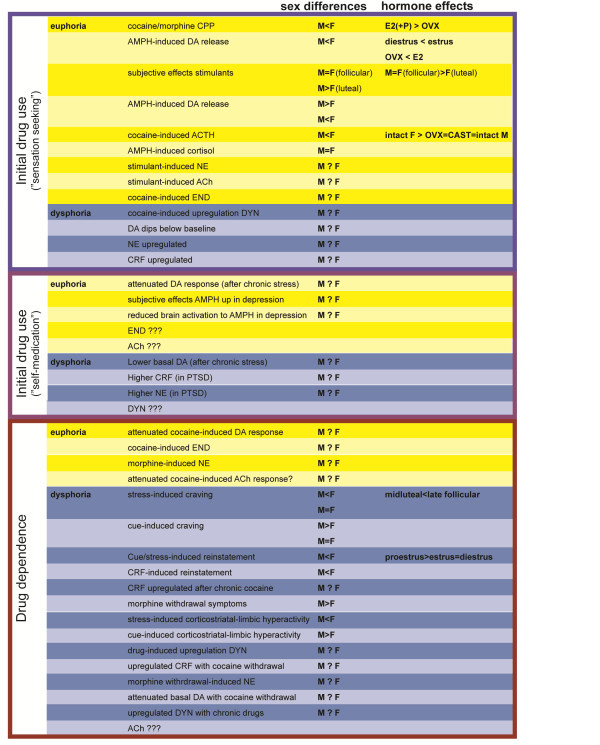
**Sex differences and the influence of gonadal hormones in the effects of abused drugs.** The contents of this figure complement Figures 1 and 5 and emphasize sex differences (when known) and hormone effects (when known) in the effects of drugs of abuse (as depicted in the figures) with initial drug use without preexisting psychopathology (euphoria-seeking), initial drug use when using as self-medication, and during dependence. Yellow shading indicated changes associated with euphoria/positive effects, and blue shading indicated effects associated with dysphoria/negative symptomatology (M: male, F: female, for other abbreviations see list).

In this review, we will primarily be focusing on sex differences in the DS/NAc and CeA/BST, as these regions are linked to the positive reinforcing effects of drugs, patterns of drug use and the negative experiences associated with withdrawal [147]. Neurotransmission within the DS/NAc and CeA/BST is influenced by DA and NE signaling originating primarily from within the substantia nigra pars compacta (SN), ventral tegmental tegmental area (VTA), locus coeruleus (LC) and nucleus of the solitary tract (NTS), which is largely thought to underlie the effects of drugs on motivation, attention and affect (Figure 2). While these brain regions contain multiple classes of local and projection neurons with diverse phenotypes, we will be focusing on DA, NE, the endogenous opioids (e.g., dynorphin, DYN and enkephalins, ENK), CRF, and ACh, as there is widespread evidence of basal sex differences in these neurochemical systems (Figure 3), which may become altered/exacerbated by drugs and contribute to sex differences in the development of addiction (Figure 4).

The GABAergic medium spiny projection neurons (MSN) in the NAc and DS comprise one of the primary sites of integration for cortical, thalamic and limbic afferents, and in turn exert enormous control over voluntary actions and the reinforcement of motivated behaviors. MSN are segregated into two major classes based on the their patterns of axonal projections (i.e., the “direct” striatonigral and “indirect” striatopallidal pathways), electrophysiological and neurochemical properties, and receptor profiles [148-152]. The striatonigral MSN preferentially express D1 receptors, dynorphin (DYN) and substance P, whereas striatopallidal MSN preferentially express D2 receptors, adenosine 2A receptors and enkephalin (ENK). There is also a sub-population of “mixed” MSN that co-express D1 and D2 receptors, DYN and ENK and send projections in both the striatonigral and striatopallidal pathways; however, the exact roles of these neurons in basal ganglia function are still being clarified [153,154].

The dynamic opposition of striatonigral and striatopallidal MSN is thought to underlie their contributions to action selection [155]. Striatonigral MSN disinhibit downstream motor circuits (hence there being called “go” neurons), whereas striatopallidal MSN generally inhibit motor activation (hence the term “no go” neurons). Striatonigral and striatopallidal MSN in different subregions of the striatum (e.g., DS and NAc) receive unique patterns of inputs from different cortical, thalamic and limbic regions, and in turn modulate distinct aspects of action selection and form specific processing loops for motor, limbic and cognitive processing [156,157].

The distinct neurochemical and receptor profiles of the striatonigral and striatopallidal MSN has enabled the generation of transgenic mice in which each pathway can selectively be activated or silenced (reviewed in [158,159]). These models have provided experimental validation of basal ganglia models of action selection, as well as providing insights into how drugs of abuse influence behavior. In general, it appears that striatonigral MSN activity is essential for conditioning appetitive responses for both food and cocaine rewards (i.e., CPP) and cocaine sensitization of locomotion, whereas striatopallidal MSN activity is required for aversive conditioning and behavioral inhibition (e.g., attenuation of cocaine sensitization and AMPH and cocaine CPP) (Figure 2) [160-164].

In addition to their effects on basal ganglia structures and action selection, drugs also exert a tremendous influence over neural circuits regulating motivated behaviors (e.g., ingestive, reproductive and defensive behaviors), endocrine systems and the autonomic nervous system [165-168]. The CeA and BST are two of the more prominent structures within these circuits, which have been implicated in the effects of drugs on emotional reactivity, stress responses and affective state (Figure 2) [145,169].

The exact roles of the CeA and BST in these and other processes are still being clarified [170,171]. Nevertheless, certain populations of neurons, identified by their neuropeptide transmitters (e.g., CRF, DYN and ENK), often function in opposition to one another to fine-tune the output of these systems and coordinate emotional, endocrine and physiological responses to various stimuli, including drugs.

In general, the ENK neurons function as mediators of positive affect, inducing positive feelings of calm, euphoria and contentment, and promoting active stress-coping responses and recovery from stress [172-175]. Conversely, the CRF and DYN neurons in the CeA and BST participate in the generation of negative affect, inducing negative feelings of dysphoria, aversion and anxiety, and promote passive coping mechanisms and activation of stress responses [176,177]. The function of these systems are normally integrated and balanced to produce the full spectrum of affective behaviors and homeostatic responses required to navigate life’s daily frustrations and joys. However, repeated drug exposure induces plastic changes in many of these systems, which result in their dysregulation in the absence of drugs and contributes to the psychological and physical symptoms of withdrawal. Additionally, activation of these neurons by stress and conditioned stimuli (i.e., drug-paired cues and environments) may trigger anticipatory changes in affective state and homeostasis that contribute to drug craving, seeking and use [145,178,179].

In the following sections, we will discuss the roles of DA, NE, CRF, DYN, ENK and ACh in regulating the functional output of the DS/NAc and the CeA/BST and how sex differences in these systems may contribute to the different profiles of subjective effects, intake and sequelae of withdrawal in males and females.

### Dopamine

#### Dopamine neurotransmission in the NAc/DS

The activity of DA neurons in the SN and VTA ranges from regular pacemaker firing to burst firing, which translate into tonic and phasic patterns of DA release that determine extracellular DA concentrations in the NAc and DS [180]. Stimuli with rewarding or salient features (or that predict rewarding stimuli) induce brief bursts of DA activity, whereas aversive stimuli typically inhibit the firing of DA neurons and reduce DA concentrations [181-183]. The dynamic patterns of extracellular DA concentrations resulting from these changes in activity are interpreted by MSN through the differential activation of D1 and D2 receptors located primarily in striatonigral and striatopallidal circuits, respectively [150,184].

All drugs of abuse increase DA concentrations in the NAc and/or DS [185,186]; however, their mechanisms of action can be quite different. The rapid increase in DA activates D1 receptors and biases the output of the striatum towards the striatonigral pathway. Repeated drug exposure induces DA-dependent plastic changes in striatonigral circuits that mediate their reinforcing effects on behavior (e.g., CPP and sensitization of locomotion).

The excessive extracellular DA concentrations induced by repeated drug exposure engage compensatory mechanisms that function to constrain tonic DA neurotransmission. Thus, reductions in basal DA concentrations in between periods of drug use lead to reduced D2 receptor occupancy and preferential activation of striatopallidal MSN, which may underlie the aversive (or anhedonic) state characterizing these withdrawal periods [187]. There is even evidence that D2 receptor expression is reduced following chronic drug exposure, which may be an additional mechanism to restore balance between the striatonigral and striatopallidal circuits.

Extracellular DA concentrations in the NAc and DS are regulated by the clearance of DA from the synapse by the DA transporter (DAT), which is also one of the major targets of the psychostimulants. Cocaine increases DA concentrations by blocking the DAT, and chronic drug use results in increased DAT levels and function that are thought to contribute to craving, withdrawal-induced anhedonia and binging. DAT activity was increased in the DS and NAc during withdrawal from cocaine self-administration, although there were region-specific changes in trafficking and signaling pathways regulating DAT activity [188].

While the activity of DAT appears to be increased after withdrawal from cocaine self-administration, its sensitivity to cocaine (but not AMPH) is actually reduced [189,190]. These changes in DAT activity and cocaine sensitivity are associated with reduced DA release, which could play a role in the development of drug tolerance, and suggest that the ability of DAT to regulate extracellular DA concentrations and serve as a drug target can be dissociated.

### Sex differences in DA neurotransmission in the NAc/DS

The number of mescencephalic DA neurons has been reported to be sexually dimorphic in many species. In rats, males have more DA neurons in the SN and females have more neurons in the VTA [191-193], whereas in non-human primates, females are reported to have more neurons in the SN than males [194]. DA neurons in the SN often send collateral axons to multiple cortical and subcortical areas, whereas VTA DA neurons primarily innervate a single target [195,196]. Thus, the sex differences in the relative number of DA neurons in these areas may have functional implications in terms of connectivity and integration between cortical and subcortical processing. These sex differences aren’t observed in all laboratory rodent strains [197], which is consistent with the intrinsic variability of the SN/VTA DA neurons and response to DA manipulations across strains of mice and rats [198-201].

Differences in the number of DA neurons are influenced by several factors, including sex chromosome complement, the presence of the *sry* gene [192,202,203] and gonadal hormones [204,205]. Gonadal hormones also regulate the density of DA terminals in many brain regions, including the NAc and DS [206-214]. The effects of gonadal hormones on DA terminals in these regions are due in part to intrinsic effects within DA neurons, many of which contain estrogen receptors (primarily ERβ) and androgen receptors (AR), as well as extrinsic effects (e.g., signaling in glia and neuronal afferents) that impinge upon DA terminals and cell bodies [215,216].

Interestingly, subpopulations of DA neurons that project to discrete brain regions appear to have unique profiles of steroid receptors, suggesting that gonadal hormones might selectively influence regional DA neurotransmission to affect certain aspects of behavior [209-212,217,218]. For example, DA neurons projecting to dorsolateral DS, which is involved in sensorimotor integration, are largely devoid of ERβ and AR, whereas DA neurons projecting to associative regions of the DS express ERβ and those projecting to the NAc primarily express AR [219]. Clearly steroid receptors within DA neurons are only one avenue through which gonadal hormones can influence DA neurotransmission in the NAc and DS, as demonstrated by the robust increase in D2 receptor binding in the dorsolateral DS following E2 treatment, even though the DA neurons projecting to this region lack ERβ and AR [219-221].

Beyond differences in DA neuron number and terminal density, there are likely sex differences in the firing rates of DA neurons, which could impact extracellular DA concentrations in the NAc and DS. The firing rate of DA neurons in the VTA fluctuates over the course of the estrous cycle, with generally higher firing rates and more burst firing in estrous and diestrous females relative to those in proestrus [222]. The reduction in firing rate during proestrus may be caused by increased negative feedback on DA neurons (e.g., D2 autoreceptors) induced by E2 [222]. Additionally, the effects of E2 on sensitivity to DA negative feedback varies in different subpopulations of DA neurons, with some showing increased sensitivity and others reduced sensitivity [223], which may reflect DA neurons projecting to different brain regions. E2 has also been shown to alter the firing rate of DA neurons in the SN of both males and females, with both increases and decreases in activity, as well as a general synchronization of firing patterns [224]. Thus, gonadal hormones are likely to influence DA firing patterns in both sexes, with more dramatic fluctuations in females over the course of the estrous cycle.

There is evidence from microdialysis studies that extracellular DA concentrations also vary over the course of the estrous cycle and between GDX males and females, which may contribute to sex differences in the function of the striatum [225,226]. In the DS, basal extracellular DA concentrations are significantly lower in diestrous females compared to both males and proestrous/estrous females, which are not significantly different from one another [226]. There are also indications that women have greater basal extracellular DA concentrations in the DS relative to men [227]. Other studies in humans, however, report no sex differences in basal DA concentrations, which is also consistent with the preclinical data (i.e., sex differences only during certain phases of the reproductive cycle).

DAT expression and function change over the reproductive cycle, and following GDX and hormone replacement, which may contribute to sex differences in extracellular DA concentrations [228-232]. In intact females, DAT binding in the DS is greater during diestrus compared to proestrus [230], which fits with the pattern of extracellular DA concentrations [226] (i.e., lower DA with increased DAT binding, and higher DA with decreased DAT binding). In OVX females, E2 increases DAT binding to levels of intact females [229]. Post-menopausal women also show increased DAT binding following E2 replacement therapy [233], which supports the idea that exogenous E2 can positively regulate DAT function in an E2-deprived condition, whereas it may normally function to suppress DAT function (and thereby increase DA neurotransmission) in the context of cycling hormone levels.

Fast scan cyclic voltammetry measurements of DA in the DS indicate that females have greater release and uptake parameters, relative to males, and that these do not fluctuate over the estrous cycle [234,235]. At low frequency stimulation, the greater uptake potential of females matches their greater release, such that DA transients are similar between the sexes; however, at higher stimulation frequencies reuptake is unable to match release producing larger evoked DA transients in females. Greater DA synthesis capacity and DAT availability have also been reported in women, relative to men [231,232,236], suggesting that sex

Collectively, these data suggest that many factors contribute to sex differences in DA function within the DS and NAc. While the absolute levels of extracellular DA concentrations in these regions might only be different during certain phases of the reproductive cycle, the temporal patterns of DA tone in males and females are quite distinct. All evidence in males suggests that their DA tone in the DS/NAc is relatively stable from day to day, which may promote a consistent balance between the output of striatonigral and striatopallidal MSN under basal conditions. The fluctuating DA tone of females, suggests that the balance between striatonigral and striatopallidal MSN may also be shifting over the reproductive cycle, with greater striatopallidal dominance during periods of low DA (i.e., diestrus and OVX). Thus, the ability of drugs to engage striatonigral circuits would face variable opposition by striatopallidal circuits depending upon the stage of the reproductive cycle.

In light of the many sex differences in these DA systems, it is not surprising that DA function is differentially affected by drugs in males and females. Sex differences in DA release to psychostimulants have been reported for humans as well as animals. Munro et al (2006) reports that men show greater DA release in the NAc and greater subjective effects to AMPH compared to women [237]. In contrast, greater DA release to AMPH has been found in women in the globus pallidus as well as striatal and cortical regions [238]. Neither study controlled for menstrual cycle, so differences could be due to the use of different tracers, regional variation among brain structures, menstrual cycle effects, as well as a combination of these factors.

Female rats show greater DA release in the DS than males in response to electrical stimulation of the medial forebrain bundle and cocaine [234,235]. Many sex differences in the effects of drugs on DA function are the result of gonadal hormones. AMPH-induced striatal DA release fluctuates over the estrous cycle of rats, with an augmented response during the evening of behavioral estrus compared to diestrus [92]. Administration of E2 (but not P) to OVX female rats increases rotational behavior and AMPH-induced DA release in the DS [225,239,240]. It is to our knowledge unknown if similar sex-differences and effects of E2 on morphine-induced DA release occur.

Sensitization of the DA response to repeated psychostimulant and opioid exposure has been found in preclinical studies, however clinical studies rather show the occurrence of tolerance, as in diminished positive effects of drugs and a blunted DA response to drug exposure in addicts [22,241-243]. This hypo-dopaminergic state is believed to contribute to anxiety and persistent drug seeking in drug-dependent subjects [242,244]. Chronic exposure to cocaine has been found to attenuate both baseline and cocaine-induced DA levels in the NAc of rats ([189,245-247], but see [248]). Sex differences have been reported for sensitization to psychostimulants in rodents, which is likely related to augmented DA responses [249,250], with females showing a higher level of sensitization than males. E2 increases sensitization in OVX females [251-253], whereas the effects of CAST and T replacement are more variable [249,252,254-256]. We are unaware of any studies investigating sex differences in tolerance. One could speculate that tolerance develops faster in women than in men, resulting in more rapid escalation of drug use to compensate for the attenuated positive effects of the drugs.

### DA neurotransmission in the CeA/BST

DA neurotransmission in the NAc and DS is essential for shifting the balance between striatonigral and striatopallidal circuits; however, the role of DA in the CeA and BST is less clear. The monoaminergic innervation of the CeA and BST includes both DA and NE projections, with discrete subnuclei in each region preferentially receiving inputs from either DA or NE neurons, or both. Within the dorsolateral BST (a region that primarily receives DA projections), intra-oral delivery of sucrose rapidly increases, whereas quinine delivery reduces, extracellular DA concentrations [257]. Reinforcing drugs also increase extracellular DA concentrations in the BST [258]. Thus, DA signaling is likely to bias neurotransmission in the CeA and BST towards outputs that promote positive arousing, emotional and affective responses. This is also supported by the ability of DA infused into the CeA to reduce the number and severity of gastric ulcers following stress [259-261], which may involve interactions with the ENK and CRF systems [262,263].

The ability of DA in the CeA/BST to modulate affective responses are partially due to its effects on inhibitory neurotransmission within these brain regions. DA attenuates evoked inhibitory synaptic currents in the CeA and BST, through the activation of presynaptic D2 receptors [264-266]. Cocaine self-administration (but not yoked non-contingent administration) modifies this effect, such that DA subsequently increases inhibitory currents through a D1-dependent mechanism that persists during withdrawal [266].

It is difficult to assign these effects of DA and cocaine to discrete neurons and projection pathways, due to the numerous types of neurons in the CeA and BST [267,268]. It does suggest, however, that the neural systems initially disinhibited by drug-induced DA signaling that contribute to the rewarding effects of cocaine (ostensibly ENK in our model) eventually become inhibited by drug-induced adaptations in D1 receptor signaling pathways [266].

### Sex differences in DA neurotransmission in the CeA/BST

There have been few rigorous studies of sex differences in DA function in the CeA and BST, especially since many studies that rely on micro-dissection techniques include additional nuclei with very different developmental origins, cell types and signaling molecules (e.g., basolateral amygdala). In gross dissections of the amygdala, males and females have similar tissue DA contents, whereas males have much greater DOPAC concentrations, suggesting that the kinetics of DA neurotransmission might be sexually dimorphic [269].

There are not many studies that have examined sex differences in DA receptor expression in the CeA and BST. Interestingly, a greater proportion of D3-containing neurons in the amygdala also co-express D1 and D2 receptors in females, relative to males, a pattern that emerges after puberty [270]. Thus, the activation of DA receptors in the CeA/BST may induce very different signaling cascades in males and females.

### Norepinephrine

Nearly all drugs of abuse increase NE concentrations in several brain regions, including the Fcx, DS, NAc, BST and CeA [271-274]. The increases in extracellular NE concentrations can occur acutely in response to the drug (thereby contributing to the initial positive or negative drug effects), develop over the course of chronic drug exposure (thereby contributing to the transition to compulsive use and habit formation), or manifest during withdrawal (thereby contributing to craving and negative reinforcement processes).

The origins of NE afferents are located in the LC (A4 and A6 cell groups), the dorsomedial medulla (e.g., the nucleus of the solitary tract, NTS, or A2 cell group) and the rostroventral medulla (RVM, or the A1, A5 and A7 cell groups) [275]. Due to their specific patterns of inputs and outputs, the different groups of noradrenergic neurons have distinct, albeit overlapping, roles in drug use and the development of addiction.

The dynamic contributions of noradrenergic signaling to early and late phases of addiction, as well as to the positive and negative effects of drugs [276] are likely due to several factors, including the multitude of NE cell groups, their diverse array of overlapping and unique afferents and projection pathways (e.g., dorsal vs. ventral noradrenergic bundles) and their reciprocal interactions with other neurochemical systems (most notably CRF and the endogenous opioids) [277-280].

Knowledge about the role of the noradrenergic system in the effects of drugs of abuse comes mostly from manipulations of this system and subsequently investigating effects of drugs of abuse. The loss of the α1b-AR results in the attenuation of locomotor activation and sensitization to AMPH, cocaine and morphine [276,281]. It also inhibits morphine CPP and reduces oral intake of cocaine and morphine in a 2-bottle choice test. All of these effects are manifested in α1b-KO mice even though post-synaptic DA signaling appears to be unaffected in the NAc and DS [276]. The endogenous ligand mediating the effects of these drugs through the α1b-AR is unknown (i.e., either DA or NE). Dopamine β-hydroxylase (DBH) knockout mice do not exhibit CPP for morphine or cocaine, even though they demonstrate CPP for food rewards [282]. DBH knockout mice also fail to show increased anxiety following acute cocaine administration, as indexed in the elevated plus maze [283]. Both of these deficits are corrected following restoration of NE biosynthesis, suggesting that they are the result of the NE deficiency, as opposed to developmental changes in the underlying neural circuitry of reward and aversion. Additionally, pretreatment with disulfiram (an inhibitor of DBH, among other enzymes) or propranolol (a non-selective β-adrenergic receptor antagonist, βAR) attenuates the acute anxiogenic effects of cocaine in wild type mice [283]. Systemic propranolol also reduces cocaine self-administration, which may reflect its potentiation of DA overflow in the NAc and putative increase in inter-infusion interval [284]. Systemic treatment with prazosin (an α1 antagonist) attenuates the motivation for cocaine in rats trained under long-access conditions, whereas α2 or β1 antagonists are ineffective [285]. In this same study, rats under long-access conditions had significantly fewer α1 adrenergic receptors in the bed nucleus of the stria terminalis compared to animals under short-access conditions or drug-naïve rats.

In addition to these reinforcing effects, NE systems also contribute to the aversive effects of drugs, especially during periods of withdrawal. Many of the negative consequences of withdrawal (behavioral aversion and physical symptoms of distress and negative affect) are attenuated following the peripheral administration of adrenergic receptor antagonists (e.g., βAR and β2AR) [286-288]. Ventral, but not dorsal, noradrenergic bundle lesions attenuate opiate withdrawal-induced aversions, but neither lesion attenuates the physical symptoms of withdrawal [287].

### NE neurotransmission in the NAc/DS

There are several mechanisms potentially contributing to the role of NE in the positive, or reinforcing, effects of abused drugs, which all primarily relate to the modulation of DA neurotransmission in the striatum (especially the NAc). The overall effects of NE manipulations on striatal DA concentrations are the product of both local effects within the striatum. The systemic administration of propranolol potentiates cocaine-induced DA overflow in the NAc, which is associated with enhanced locomotion [284]. The local effects of NE within the striatum are more complex. In general, activation of βAR within the NAc increases extracellular DA concentrations, whereas activation of α2AR (most likely α2A-AR) reduces NE concentrations, without affecting DA concentrations [289]. Little is know about sex differences in the effects of stimulants or opioids on striatal NE signaling. There appear to be no sex differences in basal levels of NE in the NAc and DS [290]. While DS tissue from males showed a greater AMPH-induced NE release than females during most stages of the estrus cycle [291].

### NE neurotransmission in the CeA/BST

The BST may be the site of action for the aversive effects of NE during withdrawal, as β-adrenergic receptor (βAR) antagonists attenuate the aversion and some of the physical symptoms of withdrawal when infused into this region [287]. The infusion of α2AR agonists into the BST also reduces aversion and some of the physical symptoms of morphine withdrawal, which may be related to the negative regulation of NE release by α2AR autoreceptors [287]. In animals addicted to opiates, the BST is activated during precipitated withdrawal, and selective β-adrenergic antagonists attenuate this response [288]. In addition, lesions of the ventral noradrenergic bundle that sends projections to the CeA/BST and NAc, unlike lesions of the dorsal bundle that target the Fcx, attenuate withdrawal-induced conditioned place aversion [288]. Infusions of βAR antagonists into either the BST or CeA attenuate stress-induced reinstatement of cocaine seeking [292].

The activation of the BST may be downstream of increased NE release, as chronic morphine treatment increases extracellular NE concentrations in the BST, which is further increased during withdrawal [274]. Chronic cocaine self-administration alters noradrenergic signaling within the CeA/BST, including up-regulation of the NE transporter [293,294]. Finally, blockade of α2aAR or β1AR within the CeA prevents the development of conditioned place aversion following systemic treatment with acetic acid, even though animals still display the physical signs of pain [295]. NE levels in amygdala do not differ by sex [296,297], it is not known if there are sex differences in drug-induced changes in NE signaling in the BST and CeA.

### NE neurotransmission in the LC/NTS

Chronic morphine exposure robustly increases TH gene and protein expression in the LC [298]. This may be related to the hyper-activation of LC neurons following drug-induced adaptations in ENK, DYN, CRF and glutamate signaling [299-302]. Conversely, chronic cocaine administration reduces TH-immunoreactivity in the LC and NE transporter-immunoreactivity in the olfactory bulb (a target of LC noradrenergic neurons) [303].

The LC has long been recognized as a sexually dimorphic structure, both in terms of volume, neuron number and cellular morphology [304-306]. LC and NTS both contain estrogen receptors (both ERα and ERβ) and androgen receptors [307-309]. Thus, gonadal hormones regulate the activity of LC neurons and NE biosynthesis in the LC and NTS (e.g., expression of TH, GTP cyclohydrolase (GTPCH), and DBH) [309-312]. Sex differences in TH expression have largely been attributed to the sex-specific patterns of ERα and ERβ, with males having more ERα and roughly equivalent ERβ expression relative to females [309]. The different patterns of ERα and ERβ may also underlie the sex-specific responses to gonadectomy, which increases and decreases TH expression in the LC of males and females, respectively [311]. Exogenous E2 reverses the effects of gonadectomy in males and females [311], as does treatment with exogenous T or 3β-diol (an ERβ selective ligand) in males [309]. Neural activation in the LC also varies over the course of the estrous cycle, with E2 reducing activation and P reversing this inactivation [310]. Even though the noradrenergic system plays a role in the effect of drugs of abuse, and sex differences in this system are well known, there is very little research investigating sex differences in the interaction between NE and drugs of abuse.

### Corticotropin releasing factor

#### Interactions with drugs of abuse

The CRF neurons in the CeA and BST are important for mediating the emotional responses to stress and contribute to many aspects of drug abuse, including initiation of drug taking, as well as generation of a negative affective state on drug withdrawal (Figure 2). CRF neurons also contribute to the effects of stress on craving and relapse due primarily to their projections to DA and NE neurons in the VTA, LC and NTS [145,313-316]. Extracellular CRF concentrations are increased in the CeA following withdrawal from cocaine, opiates, cannabinoids, alcohol and nicotine, supporting the notion that CRF mechanisms contribute to the negative symptoms associated with withdrawal after addiction [145,176,317-319].

The CRF projections to the LC and NTS are one component of an adaptive response that increases the activity of NE neurons and contributes to increased attention and vigilance, coordinated with anticipatory physiological responses [277,320,321]. The effects of CRF on DA neurons are complex, as CRF can increase the firing rate or potentiate negative feedback mechanisms (e.g., D2 autoreceptors) that inhibit DA neuron firing [322-324]. The mixed effects of CRF on DA may reflect different effects on subpopulations of DA neurons conveying different signals (e.g., motivational salience, reward value, or a general alerting function) [325]. Collectively, the effects of CRF on DA neurons may serve to interrupt behaviors mediated by striatonigral MSN and promote the transition to striatopallidal circuits for more appropriate defensive/avoidance responses.

Cocaine exposure also induces plastic changes within the VTA and the CRF projections to the VTA that result in enhanced glutamate and DA release. While stress increases CRF release in both naïve and cocaine-experienced animals, only cocaine-experienced animals display the potentiated glutamate and DA release to result in the reinstatement of cocaine seeking [318,326]. Interestingly, CRF preferentially induces reinstatement of cocaine seeking in animals exposed to long-access, but not short-access, cocaine self-administration. Therefore, the pattern of drug intake is an important determinant of these plastic changes in CRF signaling [327]. Females appear to be more sensitive to CRF-induced reinstatement of cocaine seeking [328], and females are more sensitive to stress-induced reinstatement [329]. The vast majority of research on CRF and addiction has been done in males, nevertheless, CRF regulation of the HPA axis is largely sexually dimorphic [329-332]. Additionally, the expression of CRF in females varies over the course of the estrous cycle and is positively regulated by E2 [329,333,334]. Thus, depending upon the time of day and phase of the cycle, one could obtain CRF levels that support greater activity in males, females or neither.

#### Endogenous opioids

For the purpose of this review, we will focus on what is known about sex differences in the endogenous opioid systems in the NAc/DS and the CeA/BST. After considering these systems, we will briefly discuss additional opioid systems that are also relevant to the effects of drugs and the development of addiction, namely the ENK neurons in the rostral medulla that project to the LC/NTS [335,336] and the β-endorphin (END) and endomorphin (EM) neurons located in the hypothalamus and brainstem, which send projections to the NAc/DS, CeA/BST and spinal cord, amongst other regions (ref).

#### Opioid systems in the NAc/DS

The striatonigral and striatopallidal MSN in the DS and NAc preferentially express DYN and ENK, respectively. While these two endogenous opioid peptides are often used to characterize the MSN populations, their specific roles in local signaling and the functional output of the striatum are unclear [148]. As MSN are GABAergic, and all three classes of opioid receptors (μOR, δOR and κOR) are coupled to inhibitory signal transduction pathways [337], they likely reinforce the inhibitory actions of MSN on their projection targets [338].

MSN are also exquisitely sensitive to endogenous opioids, which may be released from their axon collaterals or peptidergic afferents (e.g., DYN, ENK, END and EM) from other brain regions [339-344]. Striatonigral and striatopallidal MSN both express μOR; however, they appear to be enriched within striatonigral MSN [345,346]. Conversely, μOR are preferentially expressed within striatopallidal MSN [345]. Thus, the striatonigral and striatopallidal MSN have unique profiles of opioid peptides and receptors that may contribute to their different patterns of activity, regulation and responses to drugs.

Self-administration or “yoked” non-contingent heroin administration increases DYN mRNA expression in the NAc shell, but not the core or DS, and has no effect on ENK mRNA abundance in any of these regions [347]. Acute cocaine or AMPH exposure reduces DYN peptide levels in the DS [348] and increases DYN mRNA expression in the DS, but not the NAc, and has no effect on ENK mRNA in either region [349,350].. Chronic cocaine or AMPH administration (either self-administered or “yoked” non-contingent delivery) increases DYN mRNA expression in the DS, but not the NAc [348,351]. Cocaine exposure also attenuates some of the effects of exogenous DYN on excitatory neurotransmission in the NAc, which could reflect adaptations in either endogenous DYN synthesis and release and/or the expression and functional coupling of presynaptic κOR in glutamatergic terminals [352].

### Sex differences in opioid systems in NAc/DS

Females have greater expression of DYN within the striatum [353,354], whereas males have higher expression of ENK [353]. These sex differences are not always apparent [355,356], which may reflect the changes in the concentrations of these peptides over the course of the estrous cycle [357]. DYN peptide levels are relatively stable across the cycle in the NAc, whereas there is a significant reduction in the DS during estrus. Conversely, the concentrations of ENK appear to be more sensitive to fluctuating hormones, as they show more robust changes in both the NAc and DS and are significantly elevated during proestrus and estrus. The differential hormone sensitivity of DYN and ENK might also explain why sex differences are found in the former even in GDX animals [354].

If the relative concentrations of DYN and ENK reflect the activity (or influence) of each projection pathway, then it suggests that the balance between the striatonigral and striatopallidal MSN may be different in males and females. Thus, striatonigral MSN activity predominates in females (as suggested by their greater DYN concentrations), which could explain the more robust locomotor responses to stimulants in females compared with males. Conversely, predominance of the striatopallidal MSN in males (as suggested by their greater ENK concentration) may contribute to their attenuated responses to stimulants relative to females.

Ovarian hormones influence the acute response of striatonigral MSN to stimulants, as OVX females treated with vehicle or both E2 and P (but not E2 or P alone) displayed increased DYN mRNA expression in the DS following a single cocaine injection [358]. Immediate early gene expression was similar in all the female groups irrespective of hormone treatment, suggesting equivalent activation of MSN by cocaine. The activity of various intracellular signaling cascades in the striatum fluctuates over the estrous cycle [359]. Thus, drug exposure may translate into different patterns of DYN and immediate early gene expression depending upon the hormonal milieu.

### Opioid systems in the CeA/BST

Within the CeA and BST, there are discrete populations of ENK and DYN neurons that are critically involved in the regulation of stress responses and affective state. In general, the ENK neurons promote a positive affective state and facilitate positive coping responses, especially following stress [172-175], whereas the DYN neurons contribute to negative affective states, especially in regards to activating stress, anxiety and fear responses and feelings of dysphoria [176,177] (Figure 2). Many of the effects of DYN overlap with those of CRF, which may be due to the extensive co-localization of DYN and CRF in many CeA neurons [176,360].

The effects of abused drugs on ENK and DYN in the CeA and BST are complex. ENK and DYN neurons in the CeA/BST are activated acutely by drugs and recruited during withdrawal [361,362]. AMPH administration acutely increases activation of ENK neurons in the CeA and BST [363]. On the other hand, chronic cocaine administration (either self-administered or non-contingent “yoked” delivery) has no effect on DYN or ENK mRNA in the CeA [351].

Self-administration or “yoked” non-contingent heroin administration increases DYN mRNA expression in the CeA, but has no effect on ENK mRNA levels in this region [347]. Morphine treatment increases glutamatergic synaptic strength in CeA neurons, which is attenuated by the activation of δOR [364]. The changes in glutamatergic signaling and δOR sensitivity in the CeA are similar to the morphine-induced loss of ENK tone in the LC [299].

Thus, excitatory neurotransmission in the CeA is normally constrained by the endogenous ENK tone derived from the BST and amygdala [365], which may confer stress resilience and positive affect [172]. Repeated drug exposure reduces endogenous ENK tone, as indicated by the increased concentrations of δOR in synaptosomal fractions of morphine-treated rats [364], which may then contributes to dysphoria and anxiety during withdrawal.

DYN neurons in the CeA/BST send projections to the NE neurons in the LC and NTS [302,314]. The DYN projections activate κOR located primarily in excitatory afferents relaying sensory information, which attenuate the phasic activation of noradrenergic neurons, without altering their tonic firing rates [366]. Thus, the attenuation of sensory inputs to the LC (and ostensibly NTS) is thought to blunt the affective responses to aversive and/or arousing stimuli without affecting general arousal, which is conveyed by the tonic firing of LC neurons [302,366]. Blunted or reduced affect is thought to promote drug use in many individuals; however, many women report using crack cocaine specifically to “numb out and start to feel nothing,” [367].

### Sex differences in opioid systems in the CeA/BST

There are extensive reports about sex differences in hypothalamic opioid peptide and receptor expression [368,369], which generally support greater numbers of ENK neurons and density of terminals in males in some brain regions and equivalent levels in others. Brain regions with sexual dimorphisms in opioid systems tend to be those that are enriched in steroid receptors and contain other sexually differentiated features (e.g., anteroventral periventricular nucleus) [368] and E2 increases hypothalamic ENK concentrations in both males and females [369].

Much less is known about potential sex differences in the endogenous opioid systems in the CeA and BST; however, these brain regions both contain high concentrations of steroid receptors and are sexually differentiated in terms of morphology and neurochemistry. DYN concentrations in the amygdala do not vary over the course of the estrous cycle, whereas ENK concentrations are reduced during proestrus, relative to diestrus and estrus [357]. Males have greater δOR expression in the medial amygdala (MeA), whereas the staining intensity within the CeA is roughly equal between males and females [370].

#### Effects of drugs of abuse

Data on how (chronic) drug use affects the opioid system in the brain are limited and even less is known about sex differences herein. Chronic drug use results in increased mRNA levels of the DYN precursor prodynorphin in the striatum and amygdala [347,371-373]. Since activation of κ-opioid receptors results in depressive-like symptoms in rats, and these symptoms are blocked by a κ-opioid receptor antagonist [374,375], this is though to be related to the negative withdrawal symptomatology. The hypodopaminergic state found after chronic drug use [187,189,243-247] could be the result of increased κ-opioid/DYN signaling, since stimulation of κ-opioid receptors decreases DA transmission ([376]; for review see [377]), and this is thought to be a compensatory mechanism to counteract the high drug-induced DA levels.

### Additional opioid systems involved in addiction

The NE neurons in the LC and NTS also receive projections from ENK neurons in the rostral medulla (e.g., nucleus paragigantocellularis and the nucleus prepositus hypoglossi), which may represent sites of action for the calming and stress buffering effects of ENK and other μOR agonists. [302,335,378]. ENK/μOR signaling appears to preferentially inhibit tonic activity of NE neurons, without affecting phasic responses [321,379-383]. The inhibitory effects of ENK/μOR signaling on LC tonic activity are in contrast to the excitatory effects of CRF on tonic activity and the suppression of phasic activation by DYN/κOR signaling [384].

The effects of ENK on neurotransmission in the LC (and ostensibly NTS) are also mediated through the activation of δOR. Many ENK neurons co-release glutamate and the activation of δOR autoreceptors is thought to constrain glutamatergic signaling [299]. Morphine exposure reduces ENK expression in the brainstem and ENK levels in the LC and NTS, which leads to an imbalance in ENK/GLU signaling. During withdrawal, the loss of ENK tone may lead to excessive GLU signaling and hyper-activation of NE neurons in the LC and NTS, which contributes to the withdrawal syndrome [299].

The effects of morphine on pain perception are mediated primarily through the activation of μOR in the midbrain, brainstem and spinal cord; however, antagonism of DYN signaling actually blocks morphine anti-nociception in females during proestrus, but not in males or diestrous females [385]. The recruitment of a DYN/κOR component to the effects of morphine in females is due to the formation of μOR/κOR dimers that are relatively rare in males and increased in proestrous females. The acute blockade of E2 signaling (either through ERα, ERβ or GPR30 antagonists or the inhibition of aromatase), as well as the overnight (but not acute) blockade of progesterone receptors, significantly reduces the amount of μOR/κOR dimers in proestrous females and prevents κOR antagonists from inhibiting morphine anti-nociception [386].

Males also possess unique features of opioid signaling in the spinal cord that may contribute to sex differences in opiate addiction. The release of the endogenous opioid, endomorphin 2 (EM2), is regulated by both positive and negative feedback systems that are activated following the binding of EM2 to μOR autoreceptors in the spinal cord. In males, the activated μOR can couple with either Gs or Gi/o, which respectively enhance and inhibit subsequent EM2 release [387]. Under normal conditions, the negative feedback pathway predominates; however, following morphine withdrawal, compensatory mechanisms kick in that shift the balance towards favoring Gs coupling and enhanced EM2 release. The negative feedback system mediated by Gi/o activation is the only functional coupling that occurs in drug-naïve and opioid withdrawn females [387]. As this mechanism involves the regulation of EM2 from its terminals in the spinal cord, it is very possible that similar regulatory mechanisms operate in the ascending EM terminals targeting the NAc and CeA/BST.

Thus, while females are able to shift DYN signals into μOR responses through the formation of μOR/κOR dimers, males can take advantage of promiscuous G protein coupling to increase the gain on EM2 signaling following withdrawal from exogenous opiates. While these sex-specific processes have only been characterized in the spinal cord, it is possible that opioid systems in other brain regions might also engage similar sexually dimorphic mechanisms. As DYN expression is increased in the striatum following chronic drug use [347,371-373], the formation of μOR/κOR dimers may promote the engagement of very different signaling pathways in females, which could exacerbate or attenuate their vulnerability to addiction.

On the other hand, many studies have demonstrated greater negative symptoms in males during withdrawal from opiates and alcohol [388,389]. Naloxone-precipitated opiate withdrawal, which blocks μOR and endogenous opioid signaling, can produce more severe symptoms than spontaneous withdrawal [388]. While males show more severe symptoms than females during spontaneous morphine withdrawal, there is no apparent sex difference during naloxone-precipitated withdrawal. Thus, both male- and female-specific compensatory mechanisms might be compromised and masked during precipitated withdrawal, whereas the robust sex difference during spontaneous withdrawal suggests more effective mechanisms in females. The different mechanisms engaged by males and females during withdrawal might underlie the sex differences in symptom severity endorsed by alcoholics and contribute to the male bias in dependence.

### Acetylcholine

#### Interactions with DA

The large, aspiny cholinergic interneurons represent less than 5% of the total neurons in the striatum, but provide the only source of ACh for the entire structure [151,390,391]. The distribution of cholinergic interneurons within the striatum is heterogeneous, with generally higher densities found in the DS and the lowest levels in the NAc [392-394]. Several other regional differences in cholinergic signaling have also been reported, suggesting an even greater complexity to the function of ACh neurotransmission across the different divisions of the DS and NAc [395-398].

Every element within the striatum is affected by changes in ACh due to the presence of nicotinic and muscarinic ACh receptors (nAChR and mAChR, respectively) within MSN, fast-spiking GABAergic interneurons, and glutamatergic and DA afferents [151,390,399-401].

ACh and DA are critical partners in regulating the functional output of MSN in the DS and NAc. This partnership is largely mediated through their reciprocal interactions, as activation of nAChR on DA terminals is a major determinant of the activity dependence of DA release [396,401], which can also impact the balance between the striatonigral and striatopallidal MSN [150]. These dynamic changes in ACh concentrations can thus either sharpen or blunt the signals (i.e., DA) conveying the motivational salience and value of rewarding stimuli.

#### Interactions with drugs of abuse

Activation of D2 and D5 receptors located in cholinergic interneurons inhibits and facilitates ACh release, respectively [397,402,403]. Interestingly, relatively low doses of AMPH infused directly into the NAc rapidly increase extracellular ACh concentrations, which return to baseline levels upon cessation of AMPH perfusion. Whereas a higher dose of AMPH decreased ACh concentrations until well after the end of AMPH infusion, at which time ACh concentrations displayed a significant rebound above baseline. The initial increase following low dose AMPH and the delayed increase following the high dose of AMPH were both blocked by pretreatment with a D1-type antagonist (presumably acting through D5), whereas the initial decrease following the high dose of AMPH was prevented by a D2 antagonist [404]. Thus the magnitude and temporal pattern of the DA response may determine the effect of drugs on ACh neurotransmission. As females generally display greater DA responses to stimulants (at least in preclinical models- refer to DA section), it is possible that drug-induced ACh profiles will be sexually dimorphic (i.e., rapid increase and return to baseline in males due primarily to D5 activation, whereas females might show an initial decrease due to preferential D2 receptor activation and a delayed rebound as D5 activation takes over). Direct evidence for this sexually dimorphic ACh response is lacking, but suggested by two studies from Sousa and colleagues [405,406].

Self-administration of cocaine has short and long-term effects on cholinergic signaling, For example it reduces Choline Acetyltransferase (ChAT) activity in the NAc (and to a lesser degree in the DS), and after 3 weeks of withdrawal ChAT activity is still significantly reduced in both of these regions. Conversely, ChAT activity is increased in the PFC during cocaine self-administration and this returns to control values during withdrawal [407]. In addition self-administered drugs induce striatal ACh release, which is greater compared to non-contingent drug delivery [408-410]. In addition while the DA response remained relatively stable during acquisition of self-administration the ACh release also seemed to be more directly associated with the acquisition of self-administration. This may reflect the contribution of additional cortical or thalamic glutamatergic inputs regulating the ACh response. There are indications that ChAT activity might also be reduced in the NAc and DS of methamphetamine (meth)AMPH addicts, at least in individuals using high doses [411], which seems to correspond with the preclinical data. Alterations in cholinergic activity in the caudate are also reflected by increased vesicular ACh transporter immunoreactivity in high dose methamphetamine users [412].

Ablation of cholinergic neurons in the NAc, ventral pallidum and diagonal band shift the dose response curve for cocaine self-administration down and to the left, suggesting an increase in its reinforcing effects [413]. While these data suggest ACh neurotransmission is important in regulating cocaine self-administration, the exact locus of its effects are unknown, as the lesions preferentially targeted the ventral pallidum and diagonal band and had modest effects on cholinergic markers in the NAc. More selective lesions of cholinergic interneurons in the NAc, increase the sensitivity to both morphine and naltrexone-precipitated withdrawal [414]. The systemic administration of Acetylcholine-esterase (AChE) inhibitors, which increase central ACh concentrations, reduces morphine CPP in mice with intact cholinergic interneurons, but not lesioned mice. AChE antagonists also attenuate many of the effects of cocaine in intact mice, but not those with cholinergic lesions in the NAc, including CPP, locomotor activation and sensitization [414]. Thus, these data also suggest that ACh neurotransmission in the NAc opposes the reinforcing effects of drugs, which is consistent with the ACh-DA balance hypothesis in terms of promoting approach and avoidance [415]. It is suggested that increased striatal ACh release might contribute to the negative effects of withdrawal.

Both mAChR and nAChR are likely to contribute to the ability of drugs to condition behavior. Activation of mAChR with the non-selective agonist oxotremorine reduces cocaine self-administration, and this effect is blocked by concurrent treatment with a selective M1 antagonist [416]. Whereas antagonism of nAChR (mecamyline) reduces cue-induced cocaine craving in dependent subjects [417].

#### Sex differences

The preceding descriptions of cholinergic function in the striatum have largely been derived from research in males. There is however an extensive body of literature demonstrating sex differences in cholinergic function in the cortex and hippocampus [418-424], and many of these differences are also present in the striatum. We are unaware of any studies that have directly compared the effects of drugs on ACh neurotransmission in the striatum of both males and females, especially in relation to their reproductive hormone status. Sex differences in cholinergic function in the striatum are likely to contribute to differences in the effects of abused drugs.

There is some circumstantial evidence that the cholinergic systems of males and females respond differently to drugs. M1 and M2 receptor binding is increased in the striatum of female rats following repeated cocaine injections [405]. In male rats, repeated cocaine injections decrease M1 and M2 receptor binding in the striatum from 30 minutes up to 30 days after the last cocaine injection, including the same 24 hour time point examined in females [406]. Unfortunately, the two studies used different cocaine doses (5 and 10 mg/kg in females vs. 20 and 30 mg/kg in males); however, the striking divergence in the direction of the effects (i.e., increased in females and decreased in males), supports the contention that cholinergic signaling in the striatum may respond very differently to drugs in males and females.

Many aspects of ACh neurotransmission are sexually dimorphic (Figure 3); however, there are often conflicting data, which most likely reflects differences in the parameters under study (e.g., cell number, mRNA abundance, protein immunoreactivity, enzyme activity, receptor binding, etc.), not all of which are different in males and females. In the DS and NAc, the vast majority of studies suggest that males and females maintain different profiles of ACh neurotransmission.

Examinations of the effects of hormones on cholinergic markers have largely been confined to the contiguous groups of cells distributed throughout the medial septum, vertical and horizontal limbs of the diagonal band of Broca, and the substantia inominata (i.e., nucleus basalis of Meynert), which provide ACh projections to the cortex and hippocampus. Cholinergic neurons in these regions show reductions in ChAT-ir in GDX males and females, which are reversed by exogenous E2, P and T [425-429].

There are few studies examining the effects of sex and circulating hormone on cholinergic function within the striatum; however, the available data suggest that ACh signaling in the DS and NAc is sexually dimorphic. ChAT mRNA and activity vary over the course of the estrous cycle in the striatum, such that estrous females have significantly greater activity than males and females in most other stages of the cycle, which do not differ from one another [392,430,431]. Thus, when the cycle is not considered, males and females are often reported to have equivalent levels of ChAT activity in the striatum [432]. These differences are likely the result of the effects of E2 and P on ChAT gene expression, which interact to regulate ChAT mRNA abundance [433].

The density of AChE staining in the DS and NAc is not sexually dimorphic [434]; however, AChE activity in the striatum is reported to be greater in males [435] and increased in females following OVX [436]. Further muddying the waters of how these sex differences in ACh biosynthesis and degradation might impact the functional output of the striatum is the fact that ACh receptor profiles are also sexually dimorphic.

Males and females have similar total concentrations of mAChR binding sites in the striatum [430]. Overall, the affinity of mAChR is higher in cycling females rats compared to intact males, which is largely due to the increased affinity of females with high levels of endogenous E2. Exogenous E2 also increases the affinity of mAChR binding in OVX females [430]. Young women also have greater total mAChR binding in the striatum compared to post-menopausal women, which is attributed to E2, as post-menopausal women receiving E2 replacement therapy (ERT) have greater binding than their counterparts that have never had ERT [437]. Whether the sex differences in mAChR affinity reflect modifications of mAChR binding properties, or shifts in the differential expression of mAChR subtypes is unknown. Expression patterns of M1 and M1 receptor binding are different in males and females [392], which may very well translate into different patterns of synaptic integration in the striatum. Much less is known about sex differences in nAChR expression and function in the striatum. The mRNA expression of several α and β nAChR subunits in the SN and VTA appears to be similar in males and females [438].

Given the importance of striatal ACh neurotransmission in behavioral flexibility and conditioning and it interactions with DA signaling, it is not surprising that this system is implicated in the effects of drugs and the development of dependence (reviewed in [439]). However, the dynamic nature of the ACh signal (i.e., tonic versus burst/pause firing patterns) and sex differences throughout the system have made it difficult to ascertain the functional contributions of cholinergic neurotransmission within the striatum to the effects of drugs and sex differences herein.

### Stress-related psychopathologies in relation to drug use disorders

As mentioned earlier, there is a high level of comorbidity between drug abuse and (often stress-related) psychiatric disorders (i.e., major depression, PTSD and anxiety disorders, which have a 2–3 times higher prevalence in women), with comorbidity being associated with more psychological and social problems and poorer outcome [440-443]. Interestingly this association is especially prominent in women [15,442,443], The causal relationship between substance abuse and stress-related disorders is not an unidirectional one. In adolescence psychiatric disorders appear to precede the drug abuse problems in women [444,445].

**Figure 5:**
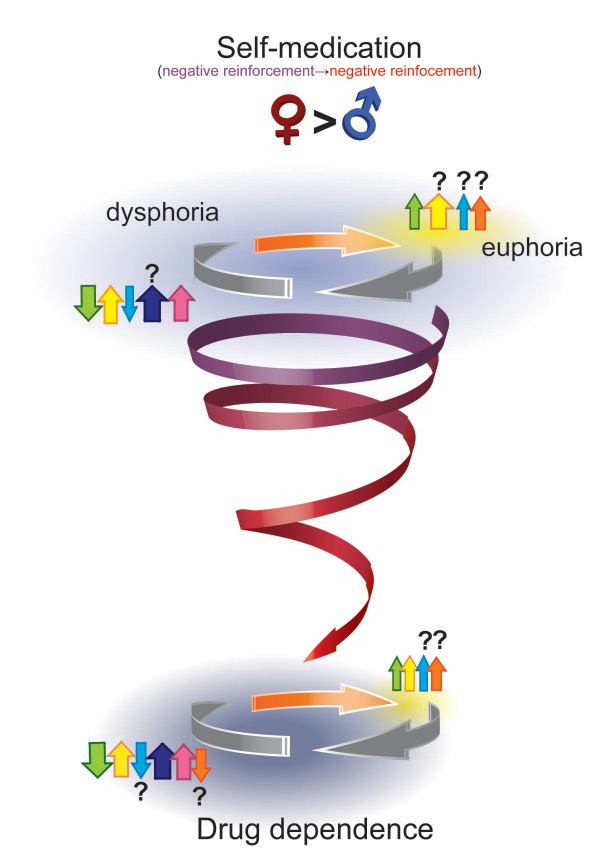
**The downward spiral from self-medication into addiction.** The spiral depicts individuals who start using drugs primarily due to negative reinforcement mechanisms, such as seeking relief from chronic negative feelings, stress-related psychopathologies or victimization (indicated by the blue/grey shading). The drug-induced (large orange arrow) euphoria is likely attenuated in these individuals and the following post-intoxication “crash,” may temporarily exacerbate their initial dysphoria that continues unabated during drug-free periods (large grey arrows). As drug use becomes less regulated, the intervals between intoxicating events become shorter (as depicted by the narrowing of the spirals), intake increases, the positive effects become further attenuated and the dysphoria/negative affective state becomes more protracted/exacerbated. These features of addiction are the result of several interacting neurochemical changes in reward-related brain regions, including a hypodopaminergic state, characterized by reduced basal and stimulated DA concentrations (green arrows), and augmented NE (yellow arrow), CRF (pink arrow) and DYN (dark purple/blue arrow) signaling. The inhibition of other opioid systems (e.g., ENK/END/EM) (blue arrow) that contribute to positive affective state may also contribute to the dysphoria experienced by those with stress-related psychopathologies and during the development of dependence. Since many of these adaptations are already present in individuals coping with chronic stress and its associated psychopathologies even before drug use, the downward spiral may be accelerated. Women are more likely to develop stress-related psychopathologies, suggesting that a greater proportion of women may initiate drug use for self-medication, whereas a larger proportion of men may initiate drug use for their positive effects (Figure 1). Sex differences in individuals with comorbid psychopathology may also lead to different trajectories toward dependence in men and women, and in sex-specific neurochemical changes. (The magnitude of neurochemical responses is indicated by the relative sizes of the arrows, refer to text for details on sex differences).

For depression, in men substance abuse frequently precedes depression, whereas in women depression precedes substance abuse [442]. A subpopulation of substance abusers begin using drugs primarily as self-medication, entering the spiral at the negative reinforcement segment. Cessation of drug taking brings on additional negative symptoms during withdrawal, in addition to the resurfacing of the pre-existing condition the drugs were taken to alleviate. This results in greater and more rapid escalation of drug use. It also puts subjects at greater risk of relapse, since drugs of abuse likely have not taken away the initial reasons that they started using drugs in the first place. Thus, the downward spiral is accelerated, again consistent with clinical and basic data discussed above (Figure 5).

With the extensive sex-differences in both the effects of stress and drugs of abuse, it is likely that stress-systems are involved in the higher vulnerability of females to certain aspects of substance abuse. Additionally, the coexistence of stress-related disorders and substance abuse could differentially affect the underlying neurobiological mechanisms in males and females.

Koob and Moal [1] depicted the process of addiction as a downward spiral with initially drug taking being maintained by drug-induced euphoria, which is followed by modest periods of negative affect (i.e., post-intoxication crashes) and relatively normal function in between intoxication events. Eventually, drug-induced neuroadaptations (i.e., hyperactivation of stress and anti-reward systems) leads to prolonged periods of withdrawal/dysphoria following cessation of drug taking. During this stage of abuse, negative reinforcement begins to maintain drug taking. Finally, in the end stage of addiction, drug taking is maintained both by negative reinforcement and habit formation, which contributes to the compulsive nature of drug taking (Figure 1).

Stress-related disorders, as post-traumatic stress disorder (PTSD) and depression, lead to neurobiological changes in brain reward systems, which likely has consequences for both positive and negative effects of drugs of abuse and could thus affect the transitioning from use to abuse. For example, depressed subjects show a hypersensitive response to the rewarding effects of AMPH [446], which is associated with a relative decrease in brain activity, in a.o. the PFC and caudate putamen [447], indicative of an hypodopaminergic state. It is to our knowledge unknown if the response to psychostimulants is changed in subjects with PTSD, or if there are sex differences in the acute effect of abused drugs in people suffering from major depression or PTSD. However for subjective effects of AMPH an opposite response has also been observed in a nonclinical sample, with subjects that reported more severe life stress had an attenuated pleasant response to AMPH. Also higher stress levels were associated with a blunted striatal DA response [448]. Unfortunately no sex differences were reported.

Both major depression and PTSD, affect HPA-axis activity albeit in different ways, in general PTSD is associated with a hyperactive central CRF and NE system and blunted HPA-axis activity, whereas with major depression it varies with the subtype of depression [449] (for review see [450]. Addiction has a high comorbidity with both, but with (possibly sex-specific) differences in underlying neurobiological dysfunctions, the consequences of drugs of abuse in the brain are likely (at least partly) dependent on the comorbid disorder. Little data is available on sex-differences. Brain maturation is differently affected in boys and girls suffering from maltreatment-related pediatric PTSD, with boys showing more adverse effect than girls [451]. Also women with PTSD show and enhanced startle response, a measure for non-specific anxiety, compared to men [452], which indicate sex differences limbic brain systems in PTSD.

As described earlier stress exposure is used as an animal model for depression and PTSD, and especially depression could be viewed as being in a dysphoric state. Although the distinction between different human stress-related disorders is hard to model specifically in animal stress models. And chronic of severe stress likely induces changes mimicking aspects of both depression and PTSD in animals.

Female rats exposed to isolation stress during puberty were shown to be more sensitive to the AMPH-induced locomotor activity compared to males [453]. Chronically stress rodents show an attenuated basal DA [454-456], which is though to underlie symptoms of anhedonia. These data appear to correspond with the findings in depressed patients of increased subjective effects and a reduced response in the brain reward system [446,447].

Repeated social defeat stress in rats affects the locomotor response to stimulants, which could be related to the positive subjective effects, cocaine self-administration. Defeated animals show a faster acquisition and a higher motivation to self-administer cocaine [457-459]. Also social stress results in escalation of cocaine, but not heroin, intake [460]. In addition, chronic social stress affects the DA response to cocaine in males, although the direction of the change depends on whether the stress was continuous or intermittent [461]. In females drug-induced increases in DA levels are attenuated after chronic stress [455].

Summarizing, the link between drug abuse and stress-related psychiatric disorders is well known. Sex differences in prevalence of those disorders is also well established. There is very limited information, however, on how this affects the pattern of drug use and if the underlying neurobiology is different dependent on the comorbid psychopathology.

## Review and conclusions

Our theoretical model presented in Figure 5 is based on the evidence reviewed above demonstrating that there are sex differences in the clinical presentation of addiction and in the neural substrates contributing to addiction. The model highlights three concepts that are essential to understanding the neurobiology of addiction: 1) addiction affects many different brain regions; 2) addiction manifests as a constellation of clinical features that collectively result in the maladaptive behavior exhibited by addicts; and 3) the presence of psychopathologies prior to drug use impacts the rate of deterioration into addiction. The magnitude of involvement of individual systems is hypothesized to differ between males and females, as described in Figure 2 and discussed above. Considered together, it is clear that effective treatments for addiction will require sex/gender-specific combinations of drugs targeting the multiple systems that are dysregulated in the addicted brain, and additionally consider comorbid psychopathologies.

Females show enhanced engagement of the DA system during initial drug exposure. As discussed above there is initially greater release of DA and an enhanced sensitivity to inhibition of uptake by stimulants in females. Chronic drug use results in a hypodopaminergic state within the striatum of both sexes, which has a greater impact in females due the enhanced sensitivity to DA. Drug use and exposure to drug-related cues are associated with increases in DA release, however, reduced DA levels between periods of drug use result in a state of dysphoria and anhedonia in which interest in natural rewards and previously preferred activities is reduced. This period is associated with enhanced drug-seeking behavior as the addict seeks to reduce craving and alleviate the dysphoria. In fact, drug use may be seen as a form of self-medication to deal with the abnormally low DA levels. Chronic drug use is also associated with enhanced engagement of NE signaling within the CeA/BST and NAc, which contributes to the negative affective state, dysphoria, anxiety and irritability associated with withdrawal. In females this negative state is exacerbated due to greater noradrenergic and CRF activity.

The sexually dimorphic behavioral patterns of drug abuse are hypothesized to be due to sex differences in the neural systems mediating acquisition and escalation of drug taking behavior. The areas of the brain involved in the neural basis for motivation begin conceptually with the ascending DA systems that project from the SN/VTA to the DS, NAc, CeA/BST, Fcx. These DA projections are involved in the initiation of drug taking and according to some models the progression from use to compulsive drug use [462].

Comorbidity between drug abuse and stress-related psychiatric disorders is associated with more psychological and social problems and poorer outcome, especially in women. Brain circuitry affected by these disorders overlaps with circuitry involved the effects of drugs of abuse, which could exacerbate the effects of drugs of abuse and lead to higher risk of transitioning from drug use to dependence and a faster escalation of drug use.

### Implications for treatment

There are very few effective treatments for psychostimulant addiction, and as such frequent relapses are a hallmark of the addiction cycle [463-465]. Naltrexone and disulfiram have been shown to reduce cocaine intake in men, but these treatments are not effective in women [466,467]. It is therefore important to test possible pharmacological interventions in both males and females. Mitigating the negative effects associated with addiction, like dysphoria, anxiety and irritability will likely reduce the frequency of drug taking and the chance of relapse. The stress-axes and κ-opioid/DYN signaling are involved in mediating these withdrawal symptoms, and pharmacological interventions targeting these systems could prove to be therapeutically interesting. With women being more sensitive to stress-induced craving, and sex-differences being present in the stress system, noradrenergic projections, and in κ-opioid/DYN signaling, it is conceivable that sex/gender will affect treatment outcome of pharmacological interventions targeting these systems.

The βAR antagonist propranolol has modest effects in promoting treatment retention and cocaine abstinence, which are mostly observed in individuals with more severe withdrawal symptoms [468,469]. Thus, noradrenergic antagonism on its own may only be effective at targeting the negative affective state ostensibly mediated by increased NE signaling in the CeA/BST.

There are even fewer studies examining the efficacy of cholinergic manipulations on clinical outcomes of stimulant addiction [470]. In general, AChE inhibitors have modest effects that are most pronounced on the subjective effects of stimulants, with little effect on actual drug use [471-473]. The relatively modest (or absent) effects of AChE inhibitors might relate to the fact that they chronically elevate ACh concentrations, which can lead to desensitization and loss of nicotinic receptors or promote the non-selective activation of both M1- and M2-like receptors.

The effects of more selective cholinergic manipulations on drug use in humans have also been examined. The administration of nicotine attenuated the subjective effects of intranasal (but not intravenous) cocaine, and increased the latency for detecting the effects of cocaine and euphoria [474,475]. Pre-treatment with mecamylamine, a nicotinic antagonist, was also shown to reduce cue-induce craving in cocaine addicts [417], whereas varenicline, a partial α4β2 agonist and full α7 agonist, tended to promote abstinence and reduced the rewarding value of cocaine [476]. The effects of nicotinic agonists and antagonists may result in similar effects through tonic activation and desensitization of nicotinic, which could explain why both treatments reduce the sensitivity to drugs and their cues. Preclinical research suggests that muscarinic interventions might also be beneficial [396,416,470,477]; however, we are not aware of any clinical data on the effects of selective muscarinic treatments on stimulant addiction. In the most of the studies examining cholinergic manipulations, the majority (or in some cases all) of the participants were men; therefore, it is also difficult to say whether men and women will show similar effects.

Opiate addiction is on the rise again, especially in relation to the abuse of prescription drugs. Unintentional overdose deaths involving opioid pain relievers have increased dramatically since 1999, and by 2007, outnumbered those involving heroin and cocaine [478]. Although more men than women use heroin, young women show a higher rate of dependence to non-medically used psychotherapeutics, which include pain relievers, sedatives, stimulants, and tranquilizers. The reason for using appears to be different between men and women, with men going for the high and rush, whereas women use it more as a form of self-medication [23]. This could reflect a difference in underlying neurobiological mechanisms on which the drugs act and have implications for possible pharmacological interventions. Studies using self-administration of opioids in both males and females are few, and if chronic exposure to self-administered opioids have differential effects on the brain of males and females as would be predicted from the data presented in this review, risk factors and treatment options will also be sexually dimorphic. With discreet behavioral profiles and neurobiological substrates of cocaine and heroin addiction [479], it cannot be expected that sex-specific findings for cocaine can be extrapolated to opioids.

### Concluding remarks

Currently, preclinical research is focused primarily on examining the acute effects of stimulants and other abused drugs and how they are influenced by pharmacological interventions. While these acute responses to drugs of abuse can provide valuable information about possible mechanisms of action, they are less informative in regards to developing new pharmacotherapies for addiction. This is because chronic drug abuse induces major changes in the brain that are often different from those occurring in response to passive drug exposure. Thus, in order for pharmacological interventions to be effective, they must target what is “wrong” in the addicted brain, which will likely not respond similar to a healthy brain exposed to acute drugs of abuse. It is therefore important to investigate possible pharmacological interventions in animal models that better reflect the suite of behavioral (and ostensibly neurochemical) changes that occur following chronic drug use, especially as they relate to addiction-like criteria.

Additionally, little is known about the neurobiological consequences of chronic exposure to drugs of abuse in females. While data collected from male subjects provide important information of how the male brain copes with repeated stimulation of the reward system, it is unlikely that the female brain responds in the same way. With drugs of abuse having sex-specific effects on behavior and the brain, it is vital to test effectiveness of new treatments and underlying neurobiological mechanisms in both male and female subjects.

## Endnotes

^1^The Harrison Tax Act is the reason the Dept. of the Treasury was responsible for enforcement of drug laws until the 1969 Dangerous Substances Act was enacted.

## Abbreviations

ACh, acetylcholine; AChE, acetylcholine-esterase; ACTH, adrenocorticotropic hormone; AMPH, amphetamine; AR, androgen receptor; BST, bed nucleus of the stria terminalis; CAST, castration; CeA, central nucleus of the amygdala; ChAT, choline Acetyltransferase; CORT, cortisol/corticosterone; CPP, conditioned place preference; CRF, corticotropin releasing factor; D1,D2,D5, dopamine receptor 1, 2 or 5; DA, dopamine; DAT, dopamine transporter; DS, dorsal striatum; DYN, dynorphin; E2, estradiol; EM, endomorphin; END, endorphin; ENK, enkaphalin; ER, estrogen receptor; Fcx, frontal cortex; GABA, gamma-aminobutyric acid; HPA-axis, hypothalamic-pituitary-adrenal-axis; LC, locus coeruleus; MSN, medium spiny neuron; MAChR, muscarinic acetylcholine receptor; Nac, nucleus accumbens; NAChR, nicotinic acetylcholine receptor; NE, norepinephrine; NET, norepinephrine transporter; NTS, nucleus of the solitary tract; OR, opioid receptor; OVX, ovariectomy/ovariectomized; P, progesterone; PTSD, post traumatic stress disorder; SN, substantia nigra; T, testosterone; TH, tyrosine hydroxylase; VTA, ventral tegmental area.
